# Supplementary material for: A qualitative study to explore the experience of parents of newborns admitted to neonatal care unit in rural Rwanda
Source: PLoS One. 2021 Aug 13;16(8):e0252776. doi: 10.1371/journal.pone.0252776 (PMC8362984; doi:10.1371/journal.pone.0252776)
Supplement: S1 File — (DOCX) [file pone.0252776.s001.docx]

**Semi-Structured Interview Guide**

**WELCOME/PREAMBLE**

Thank you for agreeing to participate in this parents interview. My name is ___________________ and I am a data collector for the study exploring “The experience of Neonatal Care Unit parents at Ruli District Hospital”

The purpose of this study is to understand the experience you undergo during the hospitalization of your baby here at Ruli DH.

**Intego y’ubu bushakashatsi ni ukurushaho kumenya imibereho yanyu mugihe murwaje uruhinja rwanyu hano ku bitaro bya Ruli.**

The information gathered in this interview will be used to help us understand what experience parents and caregivers go through here at Ruli DH Neonatal Care service and give recommendations to help make or keep the experience better.

In order to remember what we talk about in this interview, our conversation will be tape recorded. We will then write a report of what we learned from talking to many people. Your identity will be kept confidential meaning that we will not use your name or other identifying information in any of the summaries of our interview today or the reports we write from our research.

Remember, this is your time and we want to hear from you. There are no right or wrong answers. Please feel comfortable to talk about your thoughts, opinions and experiences openly. The interview is completely voluntary, so also remember that you can choose to stop at any time.

Thank you again for helping us with this important project. By sharing your ideas today, we hope to improve our understandin of how we can better care for neonates, inclusive of families’ positive experience at Ruli DH and in Rwanda. Now, let's get started.

| Participant name:  **Amazina:** |  |
| --- | --- |
| Age:  **Imyaka:** |  |
| Sex (circle one):  **Igitsina:** | Male/Abagabo Female/Abagore |
| Marital status |  |
| Which number is the sick baby |  |
| Insurance |  |
| Baby’s birth Date and Time |  |
| Baby’s NICU Admission Date and Time |  |
| What is your relationship with the baby? |  |

**Warm Up**

1. Tell me what your stay in the NCU has been like.
   1. Why was your baby admitted to the NCU?
   2. What do you know about your baby’s condition?

**EXPERIENCE ON NEONATAL ADMISSION IN NCU AND POTENTIAL STRESSORS**

1. Can you describe what it was like when they told you that your baby was going to be admitted to NCU?
2. Tell me a bit about what was going on when you learned your baby was going to be admitted to the NCU?
3. Were you expecting to have your baby admitted to NCU?
4. Were there others with you at the time your baby was admitted to the NCU?
5. What do you know about the baby’s condition which led to admission into NCU?
6. Is there a way you wish they could have helped you that time?
7. After arriving into NCU, describe your life there, its environment highlighting things that you found most stressful and most relaxing there.
8. Did anything stressed you (or still does) about your baby?
9. Did anything stressed you (or still does) about the NICU environment?
10. What do you find most helpful to you, if anything, in the NCU?
11. How do you wish they would have helped you cope well in NCU

**PARENTAL INTEGRATION IN NEWBORN CARE**

1. Can you describe what a typical day is like for you on the NCU?
   1. How do you intervene in the recovery of your baby?
2. What are your responsibilities on the NCU?
   1. Probe if needed: are you responsible for feeding? Cleaning your baby? Monitoring your baby’s condition? Keeping your baby warm in kangaroo mother care (KMC)?
   2. Who, if anyone, has taught you about the care of your baby?
      1. Probe if needed: Healthcare providers? Other family members? Other moms on the unit?
3. Have you been able to access your baby anytime that you wish?
   1. Are there times when you/your spouse were held behind and not allowed to access your baby? Tell us how it happened.
   2. Are there ways you wish to help with but get held back by health care providers or others? Tell us about them.
4. Do health care providers involve you in the decisions made for your baby?
   1. If yes, how did you feel during the decisions?
   2. If no, do you wish to be involved in the decisions?

**PARENTAL EXPERIENCE ON THEIR COMMUNICATION WITH HEALTHCARE PROVIDERS IN NCU**

1. Can you tell me about the communication you have with healthcare providers during your baby’s stay?
   1. Do you feel confident to talk to healthcare providers about your worries relating to your baby during hospitalization?
   2. How, if at all, do staff encourage you to approach them?
2. Is there an active communication between you and the NCU staff on the daily condition of the baby?
   1. How has communication been with nurses? Doctors? Other staff?
   2. Tell us more.

**PARENTAL SUPPORT FROM THE HOSPITAL STAFF, FAMILY AND RELATIVES**

1. What type of support have you received during your baby’s stay in the NCU?
   1. Who has supported you the most in this hospitalization condition?
   2. Is there other support that you feel you need? What type? From whom?
2. Looking back what was satisfying about the supports you received? What do you wish could have been different?

**Closing**

1. Thank you for sharing your experience. Is there anything else you would like to share with me about your experience in the NCU?

**Remember to thank the participant for their time and sharing their story.**
