# Supplementary material for: A qualitative study to explore the experience of parents of newborns admitted to neonatal care unit in rural Rwanda
Source: PLoS One. 2021 Aug 13;16(8):e0252776. doi: 10.1371/journal.pone.0252776 (PMC8362984; doi:10.1371/journal.pone.0252776)
Supplement: S2 File — (DOCX) [file pone.0252776.s002.docx]

**Imyirondoro y’uwitabiriye ubushakashatsi**

| Participant code:  *Kodi y’uwitabiriye:* |  |
| --- | --- |
| Age:  *Imyaka:* |  |
| Sex (circle one):  *Igitsina:* | Male/*Abagabo*  Female/*Abagore* |
| Marital status  *Irangamimerere* |  |
| What is your relationship with the baby?  *Mupfana iki n’uruhinja?* |  |
| Which number is the sick child) (1^st,^ 2^nd^, 3^rd^ child)  *Umwana urwaye ni uwa kangahe?* |  |
| Do you have Health Insurance?  *Ufite ubwishingizi?* |  |
| FROM NCU REGISTER IN SAMPLING  *IBYAVUYE MU GITABO CY’AHAVURIRWA IMPINJA* | |
| Baby’s birth Date and Time  *Itariki n’isaha y’umwana yavukiyeho* |  |
| Baby’s NICU Admission Date  *Itariki uruhinja rwinjiriye mu bitaro* |  |
| Baby’s reason for admission  *Indwara yatumye umwana ahabwa ibitaro* |  |
| Baby’s NICU discharge Date  *Itariki uruhinja rwasezerewe* |  |
| Discharge outcome  *Uko umwana yatashye ameze* |  |
| Sector  *Umurenge*  Cell  *Akagari*  Village  *Umudugudu* |  |

**Semi-Structured Interview Guide (Kinyarwanda)**

Nitwa ____________________________, ndi ushinzwe kwegeranya amakuru muri ubu bushakashatsi

None ni kuwa ……….. tariki …………. Mukwa …………… 2018/2019. Ngiye kuganira n’uwitabiriye ubushakashatsi Ruli00

Rero iki ni igihe cyawe cyo kutubwira imibereho wagiriye mu bitaro. Nta gisubizo kiricyo cyangwa ikitari cyo. Uvuge wisanzuye, utubwire ibyo utekereza, ibyo watangaho inama, ndetse n’imibereho wagiriye mu bitaro, ntakibazo.

Kuganira natwe ni uburenganzira bwawe, kandi wibuke ko ushobora guhitamo kudakomeza igihe cyose ushatse.

Nanone, mwakoze cyane kudufasha muri iki gikorwa. Twizeyeko amakuru muduha azadufasha mu kurushaho kunoza ubuvuzi buhabwa impinja ku bitaro by’akarere bya Ruli, no mu Rwanda hose. Noneho mureke dutangire.

**INTANGIRIRO**

1. Ese mwatangira mumbwira uko kuba aho bavurira Impinja mu bitaro byari bimeze?
2. Kuberiki uruhinja rwawe rwahawe ibitaro?
3. Ni iki uzi ku burwayi uruhinja rwawe rwari rufite?

**UKO BIYUMVA UMWANA AGISHYIRWA MU BITARO, N’IBIKUNDA KUBAHANGAYIKISHA**

1. Ese mwiyumvise mute bababwiye ko uruhinja rwanyu rugiye gushyirwa mu bitaro ahavurirwa impinja

PROBES (if needed):

1. Mwarimo mukora iki igihe mumenya ko uruhinja rugomba gushyirwa mu bitaro?
2. Hari abandi mwari kumwe igihe uruhinja rwashyirwaga mu bitaro?
3. Ni iki uzi ku burwayi bwatumye uruhinja ruhabwa ibitaro?
4. Hari uburyo wifuza ko bakabaye baragufashije icyo gihe?
5. Nyuma yo kugera ahavurirwa Impinja, tubwire imibereho wagiriyeyo,

PROBES (if needed):

1. Hari ibyaguhangayikishije uriyo,
2. Hari ikijyanye n’uruhinja rwawe cyaguhangayikishije?
3. Hari ikijyanye n’uko ahavurirwa impinja hameze cyaguhangayikishije?
4. Ni iki cyagufashije kwiyumva neza, niba gihari, uri ahavurirwa Impinja?
5. Ni ubuhe buryo wifuza ko bakaba ye baragufashije kumenyera ubuzima bw’ahavurirwa Impinja

**URAHARE RW’ABABYEYI MU KWITA KU RUHINJA**

1. Ese ni ibihe bikorwa bya buri munsi wakoraga mu gihe wari urwaje uruhinja ku bitaro?

   PROBES (if needed):
   1. Ni iki wafashije mu kwita ku ruhinja rwawe ngo rukire?
2. Ni izihe nshingano wari ufite mugihe wari ahavurirwa impinja?

   PROBES (if needed):
   1. Niwowe wagaburiraga umwana? waramwuhagiraga? Niwowe wamukurikiranaga? Wamushyiraga uruhu ku ruhu umurinda imbeho?
   2. Haba hari umuntu wakwigishije kwita ku mwana wawe?
      1. Abaganga? Abandi banyamuryango? Abandi babyeyi bari barwaje impinja?
3. Ese wagiye ubasha kugera ku ruhinja rwawe igihe cyose uko ubishatse?

   PROBES (if needed):
   1. Ese hari igihe wowe cyangwa uwo mwashakanye babujijwe kubona uruhinja? Tubwire uko byagenze?
   2. Ese hari uburyo wagiye wifuza gufasha mu kwita ku ruhinja rwawe ariko abaganga bakakubuza? Tubwire ubwo buryo.
4. Ese hari uruhare wagiraga mu myanzuro ijyanye n’ubuvuzi bw’uruhinja rwawe?

   PROBES (if needed):
   1. Ese iyo wabigigiraga mo urwo ruhare, wiyumvaga ute?
   2. Ese wowe wifuzaga kugira uruhare mu myanzuro ijyanye no kuvura uruhinja rwawe?

**UKO ABABYEYI BAKIRAGA UBURYO BASANGIRAGA AMAKURU N’ABAGANGA B’AHAVURIRWA IMPINJA**

1. Mwambwira uko mwavuganaga n’abaganga mu gihe mwari murwaje?

   PROBES (if needed):
   1. Wumvaga wisanzuye ku baganga kuburyo wababwiraga ibiguhangayikishije bijyanye n’uruhinja rwawe igihe wari mu bitaro?
   2. Ese hari uburyo abaganga bagushishikarizaga kubegera ngo ubabwire?
   3. Mutubwire uko mwakoranaga n’aba dogiteri
2. Buri munsi mwavuganaga kenshi n’abaganga bavura impinja ku burwayi bw’umwana wanyu?

   PROBES (if needed):
   1. Ese imivuganire yanyu n’abaforomo, abaganga n’abandi bakozi yari imeze ite?
   2. Tubwire n’ibindi.

**UBUFASHA BAHABWAGA N’ABAKOZI B’ IBITARO, N’ABO MU MUURYANGO**

1. Ese ni ubuhe bufasha wabonye igihe umwana wawe yari ahavurirwa impinja?

   PROBES (if needed):
   1. Ninde wabafashije kurusha abandi bose mugihe mwari mu bitaro?
   2. Hari ubundi bufasha mwumva mwari mukeneye? Ubuhe? Ni inde mwari mubukeneyeho?
2. Usubije amaso inyuma, mu bufasha bwose mwaherewe mu bitaro, ni iki cyari gihagije? Ni iki kindi cyakabaye cyarakozwe bitandukanye?

**UMUSOZO**

1. Mwakoze cyane kudusangiza amakuru ku mibereho mwagize.

Ese mbere y’uko dusoza, hari ikindi mwashakaga kwongeraho?

Ibuka gushimira uwitabiriye ubushakashatsi!!!

**Interview Notes**
