# Supplementary material for: A qualitative study to explore the experience of parents of newborns admitted to neonatal care unit in rural Rwanda
Source: PLoS One. 2021 Aug 13;16(8):e0252776. doi: 10.1371/journal.pone.0252776 (PMC8362984; doi:10.1371/journal.pone.0252776)
Supplement: Attachment — Submitted filename: PONE-D-20-32408_R1_reviewer comments.pdf [file pone.0252776.s003.pdf]

PLOS ONE

A qualitative study to explore the experience of parents of newborns admitted to neonatal care unit in rural Rwanda

--Manuscript Draft--

**Manuscript Number:**	PONE-D-20-32408R1
**Article Type:**	Research Article
**Full Title:**	A qualitative study to explore the experience of parents of newborns admitted to neonatal care unit in rural Rwanda
**Short Title:**	The experience of parents of newborns admitted to neonatal care unit in rural Rwanda
**Corresponding Author:**	Samuel Byiringiro, MS, Ph.Dc<br>Johns Hopkins University School of Nursing<br>Baltimore, MD UNITED STATES
**Keywords:**	Prematurity; neonatology; family-centered care; neonatal care unit; Nurses; patient satisfaction; patient experience
**Abstract:**	Abstract<br><br>Background: Neonatal Care Units (NCUs) provide special care to sick and small newborns and help reduce neonatal mortality. For parents, having a hospitalized newborn can be a traumatic experience. In sub-Saharan Africa, there is limited literature about the parents' experience in NCUs.<br>Objective: Our study aimed to explore the experience of parents in the NCU of a rural district hospital in Rwanda.<br>Methods: A qualitative study was conducted with parents whose newborns were hospitalized in the Ruli District Hospital NCU from September 2018 to January 2019. Interviews were conducted using a semi-structured guide in the participants' homes by trained data collectors. Data were transcribed, translated, and then coded using a structured code book. All data were organized using Dedoose software for analysis.<br>Results: Twenty-one interviews were conducted primarily with mothers (90.5%, n=19) among newborns who were most often discharged home alive (90.5%, n=19). Four themes emerged from the interviews. These were the parental adaptation to having a sick neonate in NCU, adaptation to the NCU environment, interaction with people (healthcare providers and fellow parents) in the NCU, and financial stressors.<br>Conclusion: The admission of a newborn to the NCU is a source of stress for parents and caregivers in rural Rwanda, however, there were several positive aspects which helped mothers adapt to the NCU. The experience in the NCU can be improved when healthcare providers communicate and explain the newborn's status to the parents and actively involve them in the care of their newborn. Expanding the NCU access for families, encouraging peer support, and ensuring financial accessibility for neonatal care services could contribute to improved experiences for parents and families in general.<br>Keywords: prematurity, neonatology, family-centered care, neonatal care unit, nurses, patient satisfaction, patient experience
**Order of Authors:**	Samuel Byiringiro, Masters
	Rex Wong
	Jenae Logan
	Deogratias Kaneza
	Joseph Gitera
	Sharon Umutesi
	Catherine Marie Kirk
**Response to Reviewers:**	Dear PlosONE Editor,<br>We appreciated you and the reviewers taking the time to thoroughly review this manuscript and provide us the useful feedback. Please find the point-to-point feedback to the comments made on the manuscript.

Powered by Editorial Manager® and ProduXion Manager® from Aries Systems Corporation

Comments are in black and the feedback in a different color.
The article present important and interesting data that will be useful for researchers and clinicians. However there are important methodological flaws that author should clarify to consider publication in PlosOne. Please take in consideration the commentaries of reviewer 2 and I also strongly recommend to use the COREQ checklist for reporting of qualitative studies. Please also justify and make a major revision of the results section of the manuscript.

Thank you for this feedback. We have included the COREQ checklist (on the last page of this document) with our response to comments. Other feedback to the comments can be found down below.
1.Please ensure that your manuscript meets PLOS ONE's style requirements, including those for file naming. The PLOS ONE style templates can be found at https://journals.plos.org/plosone/s/file?id=wjVg/PLOSOne_formatting_sample_main_body.pdf and https://journals.plos.org/plosone/s/file?id=ba62/PLOSOne_formatting_sample_title_authors_affiliations.pdf

We have updated the formatting throughout the manuscript to comply with the guidelines of PLOS ONE.

2.Please include additional information regarding the survey or questionnaire used in the study and ensure that you have provided sufficient details that others could replicate the analyses. For instance, if you developed a questionnaire as part of this study and it is not under a copyright more restrictive than CC-BY, please include a copy, in both the original language and English, as Supporting Information.

A semi-structured in-depth interview guide was developed for this study. We have attached the Kinyarwanda (the local language) and English versions of the semi-structured interview guide as a supplementary material.

3.In your Methods section, please provide additional information about the participant recruitment method and the demographic details of your participants. Please ensure you have provided sufficient details to replicate the analyses such as:
- the recruitment date range (month and year)
- a description of any inclusion/exclusion criteria that were applied to participant recruitment
- a table of relevant demographic details
- a statement as to whether your sample can be considered representative of a larger population
- a description of how participants were recruited
- descriptions of where participants were recruited and where the research took place.

a.Thank you for the suggestion. We have added the recruitment date and inclusion and exclusion criteria in our methodology section as recommended. Now it read as (p.8):

Parents or primary caregivers of neonates discharged from the NCU between September 2018 and January 2019 (three months prior to data collection) were included in the study. To ensure that parents and caregivers had been amply exposed to the NCU experience, we only included those whose newborns were admitted to the NCU for at least three days. While some mothers and caregivers were aged below 18 years, we excluded them since their experience tends to be unique because of stigma associated with teenage pregnancy in Rwanda [31]. The acquisition of consent for the participants aged below 18 is additionally different. To ensure diversity and a mix of experiences, we purposively stratified the samples by distance (living within and more than 1-hour walking distance from the facility), by admitting diagnosis (prematurity and other reasons), and outcome (died or alive at discharge).

b.We have also inserted a paragraph on recruitment procedure to describe how and where participants were recruited (P. 9):
The recruitment took place between December 2018 and January 2019. After acquiring the approval to access data from Ruli District Hospital leadership, we made the list of eligible potential participants and their geographical locations from the NCU

Powered by Editorial Manager® and ProduXion Manager® from Aries Systems Corporation

registry. We contacted the nearby Community Health Worker (CHW) to request permission on our behalf and book us an appointment to the potential participants' homes. On the appointment date, the interviewer was accompanied by the CHW to the potential participant's home which is where the data collection took place.
c. We have included the table of demographics

d.We have made clear the issue of representativeness among our limitations. (p.25): This study has some limitations. Most respondents in our study were women, and insights from fathers were relatively limited. The current study was conducted in a district hospital that receives support from a few non-government organizations. Not all Rwandan hospitals receive such support, thus the findings may not be generalizable to other Rwandan health facilities.

4.Please provide additional details regarding participant consent. In the ethics statement in the Methods and online submission information, please ensure that you have specified whether consent was informed.
We have inserted the consent process in the data collection tool and procedure section, now it reads as (P. 9):
The study was approved by the Rwanda National Ethics Committee (No. 107/RNEC/2019) and the Institutional Review Board for the University of Global Health Equity. Ruli
District Hospital provided approval for NCU data access and participants signed informed consent prior to the beginning of the interviews. To protect the participants'
confidentiality, we de-identified all quotes included in the results by removing the names, and details that could lead to identify the individual participant or their healthcare
provider.

5.We note that you have indicated that data from this study are available upon request. PLOS only allows data to be available upon request if there are legal or ethical restrictions on sharing data publicly. For information on unacceptable data access restrictions, please see http://journals.plos.org/plosone/s/data-availability#loc-unacceptable-data-access-restrictions.

In your revised cover letter, please address the following prompts:
a)If there are ethical or legal restrictions on sharing a de-identified data set, please explain them in detail (e.g., data contain potentially identifying or sensitive patient information) and who has imposed them (e.g., an ethics committee). Please also provide contact information for a data access committee, ethics committee, or other institutional body to which data requests may be sent.
b)If there are no restrictions, please upload the minimal anonymized data set necessary to replicate your study findings as either Supporting Information files or to a stable, public repository and provide us with the relevant URLs, DOIs, or accession numbers. Please see http://www.bmj.com/content/340/bmj.c181.long for guidelines on how to de-identify and prepare clinical data for publication. For a list of acceptable repositories, please see http://journals.plos.org/plosone/s/data-availability#loc-recommended-repositories. We will update your Data Availability statement on your behalf to reflect the information you provide.
The interview transcripts are all available should they be needed.
6.Your ethics statement should only appear in the Methods section of your manuscript. If your ethics statement is written in any section besides the Methods, please delete it from any other section.
The ethics statement is in the method section (P.9).
The study was approved by the Rwanda National Ethics Committee (No. 107/RNEC/2019) and the Institutional Review Board for the University of Global Health Equity. Ruli District Hospital provided approval for NCU data access and participants signed informed consent prior to the beginning of the interviews. To protect the participants' confidentiality, we utilized participant numbers in the transcripts instead of their names, and we de-identified all quotes included in the results by removing details that could lead to the recognition of the individual participant or their healthcare provider.
7.Please include your tables as part of your main manuscript and remove the individual files. Please note that supplementary tables should be uploaded as separate "supporting information" files.

	We have placed all of the tables within the text where they are referenced.
**Additional Information:**	
**Question**	**Response**
**Financial Disclosure**<br><br>Enter a financial disclosure statement that describes the sources of funding for the work included in this submission. Review the submission guidelines for detailed requirements. View published research articles from *PLOS ONE* for specific examples.<br><br>This statement is required for submission and **will appear in the published article** if the submission is accepted. Please make sure it is accurate.<br><br>**Unfunded studies**<br>Enter: *The author(s) received no specific funding for this work.*<br><br>**Funded studies**<br>Enter a statement with the following details:<br>• Initials of the authors who received each award<br>• Grant numbers awarded to each author<br>• The full name of each funder<br>• URL of each funder website<br>• Did the sponsors or funders play any role in the study design, data collection and analysis, decision to publish, or preparation of the manuscript?<br>• **NO** - Include this sentence at the end of your statement: *The funders had no role in study design, data collection and analysis, decision to publish, or preparation of the manuscript.*<br>• **YES** - Specify the role(s) played.<br><br>* typeset	CK: Received funding from Grand Challenges Canada Saving Lives at Birth as part of the evaluation of the All Babies Count initiative.<br><br>SB: University of Global Health Equity covered the cost of Dedoose software utilized in the analysis.<br><br>The funders had no role in study design, data collection and analysis, decision to publish, or preparation of the manuscript.
**Competing Interests**<br><br>Use the instructions below to enter a competing interest statement for this submission. On behalf of all authors, disclose any competing interests that could be perceived to bias this work—acknowledging all financial support and any other relevant financial or non-	The authors have declared that no competing interests exist.

financial competing interests.<br><br>This statement **will appear in the published article** if the submission is accepted. Please make sure it is accurate. View published research articles from *PLOS ONE* for specific examples.<br><br>**NO authors have competing interests**<br><br>Enter: *The authors have declared that no competing interests exist.*<br><br>**Authors with competing interests**<br><br>Enter competing interest details beginning with this statement:<br><br>*I have read the journal's policy and the authors of this manuscript have the following competing interests: [insert competing interests here]*<br><br>* typeset	
**Ethics Statement**<br><br>Enter an ethics statement for this submission. This statement is required if the study involved:<br><br>• Human participants<br>• Human specimens or tissue<br>• Vertebrate animals or cephalopods<br>• Vertebrate embryos or tissues<br>• Field research<br><br>Write "N/A" if the submission does not require an ethics statement.<br><br>General guidance is provided below. Consult the submission guidelines for detailed instructions. **Make sure that all information entered here is included in the Methods section of the manuscript.**	The study was approved by the Rwanda National Ethics Committee (No. 107/RNEC/2019) and the Institutional Review Board for the University of Global Health Equity. Ruli District Hospital provided approval for NCU data access and participants signed informed consent prior to the beginning of the interviews. To protect the participants' confidentiality, we utilized participant numbers in the transcripts instead of their names, and we de-identified all quotes included in the results by removing details that could lead to the recognition of the individual participant or their healthcare provider.

Powered by Editorial Manager® and ProduXion Manager® from Aries Systems Corporation

**Format for specific study types**<br><br>**Human Subject Research (involving human participants and/or tissue)**<br>• Give the name of the institutional review board or ethics committee that approved the study<br>• Include the approval number and/or a statement indicating approval of this research<br>• Indicate the form of consent obtained (written/oral) or the reason that consent was not obtained (e.g. the data were analyzed anonymously)<br><br>**Animal Research (involving vertebrate animals, embryos or tissues)**<br>• Provide the name of the Institutional Animal Care and Use Committee (IACUC) or other relevant ethics board that reviewed the study protocol, and indicate whether they approved this research or granted a formal waiver of ethical approval<br>• Include an approval number if one was obtained<br>• If the study involved *non-human primates*, add *additional details* about animal welfare and steps taken to ameliorate suffering<br>• If anesthesia, euthanasia, or any kind of animal sacrifice is part of the study, include briefly which substances and/or methods were applied<br><br>**Field Research**<br><br>Include the following details if this study involves the collection of plant, animal, or other materials from a natural setting:<br>• Field permit number<br>• Name of the institution or relevant body that granted permission	
**Data Availability**<br><br>Authors are required to make all data underlying the findings described fully available, without restriction, and from the time of publication. PLOS allows rare exceptions to address legal and ethical concerns. See the PLOS Data Policy and FAQ for detailed information.	Yes - all data are fully available without restriction

A Data Availability Statement describing where the data can be found is required at submission. Your answers to this question constitute the Data Availability Statement and **will be published in the article**, if accepted.<br><br>**Important:** Stating 'data available on request from the author' is not sufficient. If your data are only available upon request, select 'No' for the first question and explain your exceptional situation in the text box.<br><br>Do the authors confirm that all data underlying the findings described in their manuscript are fully available without restriction?	
**Describe where the data may be found in full sentences. If you are copying our sample text, replace any instances of XXX with the appropriate details.**<br><br>• If the data are **held or will be held in a public repository**, include URLs, accession numbers or DOIs. If this information will only be available after acceptance, indicate this by ticking the box below. For example: *All XXX files are available from the XXX database (accession number(s) XXX, XXX.*).<br>• If the data are all contained **within the manuscript and/or Supporting Information files**, enter the following: *All relevant data are within the manuscript and its Supporting Information files.*<br>• If neither of these applies but you are able to provide **details of access elsewhere**, with or without limitations, please do so. For example:<br><br>*Data cannot be shared publicly because of [XXX]. Data are available from the XXX Institutional Data Access / Ethics Committee (contact via XXX) for researchers who meet the criteria for access to confidential data.*<br><br>*The data underlying the results presented in the study are available from (include the name of the third party*	All relevant data are within the manuscript and its Supporting Information files. Fully de-identified interview transcriptions are also available should they be needed.

and contact information or URL).<br>• This text is appropriate if the data are owned by a third party and authors do not have permission to share the data.<br><br>* typeset	
Additional data availability information:	

Powered by Editorial Manager® and ProduXion Manager® from Aries Systems Corporation

Revised Manuscript Click here to access/download;Manuscript;Manuscript.docx

A qualitative study to explore the experience of parents of newborns admitted to neonatal care unit in rural Rwanda

Samuel Byiringiro[1,2*#a], Rex Wong[2,3], Jenae Logan[#b], Kaneza Deogratias[4], Joseph Gitera[5], Sharon Umutesi[6], Catherine M. Kirk[1,2]

[1] Maternal and Child Health Department, Partners In Health/Inshuti Mu Buzima, Rwinkwavu, Rwanda

[2] Bill and Joyce Cummings Institute of Global Health, University of Global Health Equity, Butaro, Rwanda

[3] School of Public Health, Yale University, New Haven, Connecticut, United States of America

[4] Ruli District Hospital, Ministry of Health, Gakenke, Rwanda

[5] Clinical and Public Health Services Division, Ministry of Health, Kigali, Rwanda

[6] Maternal, Child and Community Health Division, Rwanda Biomedical Center, Kigali, Rwanda

[#a]Current Address: Johns Hopkins University School of Nursing, Baltimore, Maryland, United States

[#b]Current Address: Partners In Health, Boston, MA

***Corresponding author**

Email: sam6as@yahoo.com (SB)

Abstract

Background: Neonatal Care Units (NCUs) provide special care to sick and small newborns and help reduce neonatal mortality. For parents, having a hospitalized newborn can be a traumatic experience. In sub-Saharan Africa, there is limited literature about the parents' experience in NCUs.

Objective: Our study aimed to explore the experience of parents in the NCU of a rural district hospital in Rwanda.

Methods: A qualitative study was conducted with parents whose newborns were hospitalized in the Ruli District Hospital NCU from September 2018 to January 2019. Interviews were conducted using a semi-structured guide in the participants' homes by trained data collectors. Data were transcribed, translated, and then coded using a structured code book. All data were organized using Dedoose software for analysis.

Results: Twenty-one interviews were conducted primarily with mothers (90.5%, n=19) among newborns who were most often discharged home alive (90.5%, n=19). Four themes emerged from the interviews. These were the parental adaptation to having a sick neonate in NCU, adaptation to the NCU environment, interaction with people (healthcare providers and fellow parents) in the NCU, and financial stressors.

Conclusion: The admission of a newborn to the NCU is a source of stress for parents and caregivers in rural Rwanda, however, there were several positive aspects which helped mothers adapt to the NCU. The experience in the NCU can be improved when healthcare providers communicate and explain the newborn's status to the parents and actively involve them in the care of their newborn. Expanding the NCU access for families, encouraging peer support, and

ensuring financial accessibility for neonatal care services could contribute to improved experiences for parents and families in general.

Keywords: prematurity, neonatology, family-centered care, neonatal care unit, nurses, patient satisfaction, patient experience

Background

Neonatal mortality makes up nearly half of all deaths among children under five years of age [1]. It is estimated that nearly 1.9 million newborn deaths can be averted by 2025 if quality maternal and newborn care interventions were implemented [2]. Expansion of access to specialized neonatal care units (NCUs) for sick and small newborns is one of the global priorities to reduce neonatal mortality [1]. Despite the tremendous benefits of NCUs in achieving better neonatal health outcomes, studies exploring the experience of parents in NCUs have shown it to be a stressful experience for families [3–8].

While there is a large body of literature regarding parental experience in NCUs in western countries, very little is known in Sub-Saharan Africa [9,10]. A small number of studies in South Africa showed that parents often experience negative emotional feelings and various challenges in NCUs [8,11]. Many parents expressed depressive symptoms and inability to bond with their newborns [12–16]. Some newborns were abandoned in the NCU, and this disruption of parental care can contribute to inferior early child development outcomes, lower educational attainment, and poorer lifetime economic earnings [17]. However, the findings and recommendations from South Africa - a high middle-income country, are difficult to transfer to other Sub-Saharan African nations - predominantly low-income countries [18].

Between 2005 and 2015, Rwanda has documented a rapid decline in under-five child mortality from 152 to 50 deaths per 1,000 live births and neonatal mortality from 37 to 20 deaths per 1,000 live births [19]. Despite such tremendous progress, neonatal deaths still contribute to 40% of all under-five deaths in Rwanda [19]. In order to accelerate the reduction in neonatal mortality, the Rwandan Ministry of Health launched the National Neonatal Care Protocol in 2012 addressing the gaps directly related to clinical neonatal care [20] and scaled-up hospital

level neonatal care countrywide. However, other than for skin to skin care, the protocol does not integrate parents into the NCU environment [21,22]. One quantitative study found high stress score and the predominant stressors were the babies' appearance and behavior among parents of newborns admitted to NCU in an urban tertiary hospital [23].

Quality healthcare requires both the provision of evidence-based clinical care to newborns and also ensuring a positive experience of the newborn and their families [24]. High quality care cannot be fully achieved unless the experience of care meets the needs of families, which requires knowing their experiences, and designing appropriate interventions [24–26]. Accordingly, this study explored the experience of parents whose neonates were hospitalized and discharged from the NCU in a district hospital in rural Rwanda. The results of this study can add to the existing literature on the experience of parents with newborn children in the NCU in low- and middle-income countries in the sub-Saharan Africa region.

Methods

Setting

This study was conducted in the catchment area of Ruli District Hospital – a rural public hospital located in Rwanda's Northern Province. With 10 beds and 5 incubators, Ruli District Hospital's NCU also serves as a referral site for hydrocephalus surgical treatment. In 2017, there were 459 NCU admissions [27]. From 2017-2019, Ruli District Hospital received support to improve the quality of care for newborns through the All Babies Count (ABC) program implemented by Partners In Health/Inshuti Mu Buzima and the Ministry of Health.

NCUs at District hospital levels in Rwanda

NCUs at the district level in Rwanda provide preventive, curative, and rehabilitation care to the small and sick newborns, and those at risk for complications [28,29]. The majority of babies who are referred to the district level NCUs are usually those born too early and/or too small (with weight below 2.5 kg), but occasionally too big (above 4 kg); babies with birth asphyxia; and babies with risk for infection as stated in the national clinical guideline (including but not limited to babies born at home, from mothers who presented with a fever during labor or 24 hours after delivery, or rupture of membranes 18 hours before delivery) [28,30].

District level NCUs exist as a separate unit from maternity even though they work closely together. They treat newborns from both the health centers of the hospitals' catchment area and the hospital maternity. Babies born at home are automatically referred to the NCU for risk of infection, and while those born at the health centers and the hospital maternities are evaluated for any necessity to refer to them to the NCU. If transferred to the NCU, hospitals' procedures vary but at Ruli DH, the baby's mother or closest relative accompany the baby inside the NCU [28].

NCUs in Rwanda provide care at varying capacities yet the health service package for public health facilities determines that they should be able to offer supportive oxygen to babies with birth asphyxia, continuous positive airway pressure to premature and term babies with respiratory distress, antibiotic therapy, thermal regulation support, fluid and electrolytes when necessary to name a few [29]. Specialized neonatal care is usually absent at district levels and newborns who require it are sent to referral hospitals' NCUs.

Design

We used qualitative descriptive and contextual approach. Semi- structured interviews were conducted to understand the experiences of parents having a newborn hospitalized and discharged from the NCU.

Study Participants

Parents or primary caregivers of neonates discharged from the NCU between September 2018 and January 2019 (three months prior to data collection) were included in the study. To ensure that parents and caregivers had been amply exposed to the NCU experience, we only included those whose newborns were admitted to the NCU for at least three days. While some mothers and caregivers were aged below 18 years, we excluded them since their experience tends to be unique because of stigma associated with teenage pregnancy in Rwanda [31]. The acquisition of consent for the participants aged below 18 is additionally different. To ensure diversity and a mix of experiences, we purposively stratified the samples by distance (living within and more than 1-hour walking distance from the facility), by admitting diagnosis (prematurity and other reasons), and outcome (died or alive at discharge).

Recruitment procedure

The recruitment took place between December 2018 and January 2019. After acquiring the approval to access data from Ruli District Hospital leadership, we made the list of eligible potential participants and their geographical locations from the NCU registry. We contacted the nearby Community Health Worker (CHW) to request permission on our behalf and book us an appointment to the potential participants' homes. On the appointment date, the interviewer was

accompanied by the CHW to the potential participant’s home which is where the data collection took place.

Data collection tool and procedures

Interviews were conducted using a semi-structured interview guide that included questions related to the parents’ and caregivers’ feelings on neonatal admission and their experience in the NCU, the quality of communication with healthcare providers, their involvement in the newborn’s care, and the support received from the hospital staff, relatives, or others during the stay. We developed the interview guide in English and translated it to Kinyarwanda - the local language for conducting the interviews.

The interview guide was pre-tested and piloted on two parents before the actual data collection. The pilot did not lead to changes in the protocol hence its participants were included in the analysis. Two female data collectors who were not healthcare providers nor worked in the study district were trained to conduct the interviews. Husbands and wives were interviewed separately. The interviewer provided information about the study to the participants and clarified that participation was voluntary. Those who accepted to take part in the study provided written informed consent before the interview begins. The interviews took place the quiet place at the participants homes. The study participants received a gift consisting of a baby hat and a bar of soap. The Principal Investigator listened to the interviews at the end of each data collection day to determine if the saturation was reached.

Data analysis

The interviews were transcribed and translated, then coded by two independent investigators. We explored the literature on the experience of parents in typical low-income settings' NCUs and used it to create the preliminary codebook. The codebook was amended using pilot interviews. The independent coders met with the Principal Investigator to resolve any discrepancies by discussion and revision of the definition of the codes. The transcripts were coded then grouped into themes. We used Dedoose software to organize data for analysis.

Ethics

The study was approved by the Rwanda National Ethics Committee (No. 107/RNEC/2019) and the Institutional Review Board for the University of Global Health Equity. Ruli District Hospital provided approval for NCU data access and participants signed informed consent prior to the beginning of the interviews. To protect the participants' confidentiality, we utilized participant numbers in the transcripts instead of their names, and we de-identified all quotes included in the results by removing details that could lead to the recognition of the individual participant or their healthcare provider.

Results

A total of 21 interviews were conducted (Table 1). Participants were aged between 20 and 56. Nineteen (90.5%) were female participants. Seventeen (80.9%) of the participants were married and 4 (19.0%) were single. Nineteen (90.5%) had children who were alive, while 2 (9.5%) died prior to discharge from NCU. Eight (31.0%) lived within an hour walking distance to the

hospital while 13 (61.9%) had to either take the bus or a motorcycle or walk for more than an hour to the hospital from their homes. Prematurity (42.8%) and infection risk or fever (14.2%) were the most common reasons for admission. All participants had health insurance.

Four overarching themes were generated from the interviews with parents regarding their experience of having their newborn hospitalized in NCU at Ruli DH. Theme one consists of parental emotional adaptation to the newborn care in the NCU. The second theme describes the parental adaptation to the NCU environment which includes the physical environment and rules and regulations of the NCU. The third theme covers the experience of parents with other people in the NCU and the last theme discusses the Financial aspects of the care and support received at Ruli DH.

Table 1. Demographic characteristics of participants

**Characteristics**		**N (%)**
Total Participants		21
Age (n=19)	20-29	8 (38%)
	30-39	8 (38%)
	>=40	3 (15%)
	Undeclared	2 (9%)
Gender	Male	2 (9.5%)
	Female	19 (90.5%)
Newborn's outcome at discharge	Alive	19 (90.5%)
	Dead	2 (9.5%)
Marital status	Married	17 (80.9%)
	Single	4 (19.0%)
Distance to hospital	<1 hour	8 (38.0%)
	>1 hour	13 (61.9%)

Reason for admission	Prematurity	9 (42.8%)
	Low Birth Weight	2 (9.5%)
	Infection risk/Fever	3 (14.2%)
	Other*	7 (33.3%)
Health Insurance	Yes	21 (100%)

[a]Other: asphyxia, congenital malformation, jaundice, not sucking on breast, respiratory distress, cord bleeding

Theme 1. Parents' Emotional Adaptation to the newborn care in the NCU

Parents' emotional adaptation to receiving newborn care in the NCU was first characterized by a mix of negative feelings and emotions resulting from the quick roll out of events in the short time from stress of delivery to the admission in the NCU. The parents found the NCU environment to look strangely different because of the unusual equipment and the type of care newborns receive. They feared that the baby was being hurt by the different nursing and medical care and procedures.

1.1. Stress due to baby's condition

Many parents reported feeling fearful when they were informed that their newborn required admission to the NCU. They feared that the child could die and that caused them sadness, uneasiness, hopelessness, disturbance, and anxiety.

> *"There was no other way I could understand it... I didn't even feel hopeful because the baby was too premature. I was just waiting for what God was to give me. My baby only weighed 1.2 kg. I felt hopeless and felt the healthcare providers were struggling for nothing."* (Mother, age 36).

Several mothers reported a very emotional response to their newborn's admission:

> *"I felt very sad and I am telling you that I would suddenly burst into tears. I was alone with the baby always expecting to be discharged the following day. I felt disturbed, wept repeatedly, then breastfeed the baby but suddenly found myself crying."* (Mother, age 28).

> *"I cried. Other mothers used to say that no other person enters there [NCU]. I felt like it was over for me when I saw myself referred to there. I thought that I would not leave soon and I imagined that my baby would eventually die. I felt very sad, and it was hard for me to accept the situation"* (Mother, age 25).

1.2. Stress due to the visual sight of the baby and the NCU

Many parents reported negative feelings caused by the look at the different tubes and wires attached to the newborns inside the incubators, the sound of the equipment and sometimes the care provided to the newborns. While the fathers were not allowed into the incubator room part of the NCU, one father reported managing to trespass into that room and described how he felt when he saw his fifth newborn in the incubator for prematurity:

> *"I felt scared to see the machines. You say, look at these machines, and the baby is lying in them naked. I was not sure that my baby would survive."* (Father, age unknown).

Another mother shared:

> *"I was stressed because I was thinking that she (my baby) would not survive. I saw that my baby was on oxygen, and another noisy machine, I lost hope because the baby spent*

the whole week in the incubator. When I arrived at the hospital, I was so worried that my baby would not make it." (Mother, age 35).

1.3. Stress due to that fear the baby is being hurt during care

Additionally, parents feared that their newborn may be hurt by the care provided in the NCU. The fear was particularly higher when the parents thought the provider was not experienced enough (student for instance) to care for the sick baby.

> *"He [a nursing student] held it [a needle] and pierced without first looking for where the vein was, and he attempted three times. The third time, I told him to stop paining my baby."* (Mother, 32).

Theme 2. Parental Adaptation to the NCU Environment

Parents reported some regulations that were positive – such as uninterrupted access to their newborn but other regulations of no visitors, no caregivers, and no eating inside the ward stressed mothers. The procedures of always cleaning hands, changing shoes, and putting on different clothing (aprons) are a source of stress to some mothers even though some mothers appreciated them due to the resulting calmness in NCU. Caregiver's stress was worsened by insufficient induction to the NCU contributing to a lack of understanding of NCU rules and procedures.

2.1. Being part of the baby's care

Mothers reported that having full access to the newborn any time throughout hospitalization, even when the newborns were inside incubators or on phototherapy, brought them comfort. Having some responsibility for the care of their newborns, ranging from paying the bills, to bringing required materials, to feeding or bathing their newborn, improved their feelings about

the situation even though the part of responsibility was occasionally a source of stress to parents who were unable to bring fulfill all their responsibilities (Paying bills, providing newborn materials such as clothes, bringing hygiene materials and so on).

> *"The healthcare providers told us, we could go at any time to have a look at our babies. They told us to be close to our babies. We hence used to come look at our babies and [go] back whenever."* (mother, age 35).

> *"They [nurses] told me to go and breastfeed my baby anytime I wanted. I felt no problem because I was free to go and see the baby anytime."* (mother, age 22).

While mothers rejoiced in being able to access their newborns at any time, the adaptation and stay was deranged when mothers were unable to care for their newborns such as lacking breast milk. The stay was additionally unpleasant for some mothers who lacked means to follow up their own health because they were still sick when the newborns were transferred to the NCUs.

> *"What stressed me is when they told me to express breastmilk for my baby. The baby stayed in the machines on oxygen, and when it was time to start pumping breast milk for the baby, I could not get it."* (mother, age 36).
>
> *"Because of taking care of the baby while I was still sick, I became sicker and felt as if I was dead. I did not know what I was sick of but when I went to seek care I had high fever and was shivering."* (mother, age 33).

2.2. Access to their babies

The fathers were the only caregivers other than the mothers reported to have some access into the general room (Kangaroo Mother Care Unit), though they had limited visiting hours and were not allowed to touch or hold the newborns, or see them if they were in the incubators' room. Other

visitors were not allowed into the NCU. The mothers felt very sad when the newborn passed away without having the father see them.

A mother reported her feeling that the father did not see their newborn before she died:

> *"I told one of the other mothers in the NCU that I was very sad because my baby was going to die before her father could at least see her. The mother told me that I should have requested it before, and I told her that nobody advised me to ask a healthcare provider for permission so that my husband could visit my baby."* (mother, age 35)

2.3. Orientation to the NCU

Parents did not appreciate the fact that they were not provided a rigorous initial orientation so that they consciously abide by the NCU regulations. They would find themselves frustrated by the things about which they should have been informed at admission. Most of quarrels between parents and healthcare providers resulted from the tendency of mothers to bring visitors to the NCU.

> *"She [healthcare provider] asked me why I had violated the rules [allowing the father of the baby to visit]. She replied that I should not allow him in without telling her first. I told her that I had read the regulation but did not know that I should get approval from her before bringing him inside."* (Mother, age unknown).

> *"They [healthcare providers] should explain to the mother where they were taking the baby and explain the life there. For instance, after I arrived, nobody explained the rules and regulations of the place except reading it for myself. None told me the procedure of things in the unit. They should improve that."* (Mother, age 25).

2.4. Physical environment of NCU

In addition to the stressors from the NCU regulations, mothers reported the NCU interior to have low temperature which make it chilly. They even feared that the coldness they felt affected the babies as well. Additionally, the nature of the NCU care makes mothers unable to rest as many of them had to watch over their newborns day and night.

> *"Cold... ohhhh, it was too cold. Our babies were freezing and were losing weight. The room was freezing."* (mother, age 44).

Despite the many stressful aspects of the NCU, mothers reported the environment to be safe from disorder and robbery, clean and quiet which provides a sense of comfort and positive feelings. Some mothers reported appreciating the safeguard put in place to minimize visitations explaining that such measures allow the NCU environment to remain clean, and silent which they thought is good for the sick newborns.

> *"I thought the place would be smelling bad. I, however, found it different. They [healthcare providers] teach them [mothers] morning and evening to have hygiene so that even newcomers can be informed about hygiene."* (mother, age 43).

> *"I was very happy with the hygiene of the NCU. I was happy with the way they restrict the access into the Unit where there are babies born with problems. You see, allowing many people inside would cause the room of sick babies to be stuffy. They avoided that. The way they prohibit the entry of shoes from outside is good."* (mother, age 27)

> *"You enter and you can even make a prayer silently in your mind to avoid making noise. You make sure there is no noise in the NCU, no one is allowed to scream, the people inside are so quiet. It is not authorized to make phone calls or other devices that may produce noise."* (mother, 35).

Theme 3. Experiences with other people in NCU

Parents reported having both positive and negative interactions with healthcare providers, and an overall strong sense of trust for healthcare providers. Other mothers in the NCU were reported to be a consistent source of support (emotional and material) for each other.

3.1. Interactions with healthcare providers

A mix of healthcare provider attitudes, some positive and others negative, were reported by parents. They were appreciative of the healthcare providers who explained the care their newborns received, provided guidance and updates on the newborn's condition, and encouraged them to ask questions.

> *"There was one woman [healthcare provider] who used to talk to us. She would say, 'Do you have any questions?', and we often responded, 'no'. She would then ask us what she would have taught us. We were more comfortable with that healthcare provider."* (Mother, age 28).

> *"We would joke with healthcare providers and occasionally forget that our babies are hospitalized. They would come and comfort us telling us that our babies would get cured. You could notice that they were doing whatever possible to converse with us."* (Mother, age 33)

The provision of information about the findings of assessments contributed to the parent's understanding of the newborn's condition and was one key factor to the positive feelings and comfort to the mothers in the otherwise hopeless conditions.

> *"It was my first time to see such a disease [jaundice], my first time to hear of it. I had hoped that he would get well soon. After they [healthcare providers] explained the*

condition to me, I felt ensured that the child shall get cured and I had no problem." (Mother, age 22).

"When I arrived, they [healthcare providers] examined the baby, looked at her vomiting and the problem in her eyes and they comforted me saying it would go away little by little. They advised me how to position my baby while breastfeeding and it decreased gradually. Gradually as they talked to me and comforted me, I regained the mood." (Mother, age 28).

However, some healthcare providers were reported to have a bad attitude which made the NCU stay stressful and challenging for some mothers. Parents reported a negative experience of interacting with some healthcare providers who spoke in a rude tone, provided no guidance or information, and showed no empathy while requesting newborn care materials from parents.

"Whenever healthcare providers entered, I trembled because they entered speaking roughly, except that you ought to get used to it. Once, I went outside immediately after breastfeeding. A few minutes after, when I came back the baby had passed stools. The healthcare provider immediately talked to me in a rude voice. I tried to explain that I was there with my baby a few seconds before. She refused to understand and roughly said I should look after my baby at every moment." (Mother, age 22).

"They yelled at me asking me why I had not yet bought clothes for my baby. I begged and told them that I could not afford to buy the clothes because I am poor. She rudely replied that poor people do not go to seek care at Ruli hospital." (Mother, age 36).

Blaming parents for the newborn's condition was another attitude repeatedly reported by parents. In most cases, the parents reported that healthcare providers blamed them for not taking good

care of their babies or not fulfilling their responsibilities (such as providing needed materials for newborn care) hence leading to worsening of the condition.

> *"My baby had a fever and was vomiting. I called the healthcare provider who was on day duty and she said that it was because of me that I was not holding my baby well."* (Mother, age 22).

Most of the parents, including those whose newborns died prior to discharge, trusted that providers were doing their best to save the newborns.

> *" I did not decline any treatment because I could see them coming to treat my baby and I felt that because they are healthcare providers my baby would be cured."* (Mother, age 33).
>
> *"I felt that because my baby is in the hands of healthcare providers, she would be fine. They would comfort us saying that no baby would die anymore, and we felt hopeful. After that, they took an exam and the result was good and I was very happy that my baby was fine. They took care of my baby. They tried whatever possible."* (Mother, age 25).

3.1. Interactions with other parents

In addition to turning to their partners, family, and relatives for support at the time of admission and during the stay, fellow mothers in NCU provided emotional and material (food, clothes, and other consumables) support which was a great comfort for many.

> *"Other mothers are the ones who helped me feel better. When I looked at their babies and they told me that their babies were sicker when they arrived than [at] that time, I felt better."* (mother, age 27).

"There was another woman next to me, who had also given birth to twins and whose caregiver was her mother. They used to lend me materials and because she also came from far, when they would have brought them food they cooked and shared with me. I did not have people who could bring me food, so I survived because there were people of goodwill at the hospital." (mother, age 25).

Other mothers also helped explain the situation of the NCU to others when healthcare providers did not:

"We had to transmit the information to the mothers who were new and did not know these instructions because healthcare providers were not always in [a] good mood of communicating." (Mother, age 25).

"There were mothers without caregivers. They go into the eating hall to see if someone can give them food. We do share food with them because one cannot finish it. I have noticed that people help each other." (Mother, age 28).

Theme 4. Financial aspects of the care in NCU and support received

Many mothers and fathers reported that the financial burden due to NCU admission was challenging and was a stress for them. They had difficulty in paying many of the NCU requirements such as specific clothing, basins, flasks as well as the hospital fee. Despite all participants having health insurance, many could not afford the co-payment. Two interviewees reported they had to sell a portion of their land to pay for the NCU costs.

"I was wondering how I would get money to pay the invoice that was 99 times more than my salary. People told me that I must sell the land to pay medical invoices. I was scared. I was wondering how I will survive when I go home because I had already sold my land in

order to buy clothes [for the baby]. I was speechless and there were times when I did not have breast milk because of problems." (mother, age 44).

"I was stressed because of the 30,000 RWF medical bill for spending a week and three days in the hospital. I was wondering where I would get the money if I had to spend one more week in the hospital. I was confused but I was also ready to sell my house to be able to pay the medical bill." (Mother, age 34). She eventually sold her land to pay the bills.

Even sometimes when the NCU was not as expensive as expected, the thought of the potential cost caused a lot of stress.

"When we went to the hospital, we wondered how we could pay the bill. I was afraid that I would not get enough money to pay the medical bills and would be held in custody at the hospital. But, luckily, I was not charged a lot of money because I had health insurance. I did not have to sell any of my goods." (mother, age 35).

The cost of hospitalization was a major source of stress, and any forms of intermittent support they had received from others, like porridge and food received from the Catholic sisters or university students supporting the hospital were very much appreciated by the parents.

"Every morning they [healthcare providers] bring the porridge and bread. I cannot blame them for anything. The healthcare providers bring the hungry people food from the Sisters. They do whatever possible." (mother, age 36).

"The most important thing that they helped us with was the free porridge in the morning. It was very impactful because a mother cannot breastfeed the baby without eating the porridge. It is the best help they gave us. We ate the porridge happily with the peace of mind." (mother, age 25).

Discussion

Parents shared both positive and negative aspects of their experience inside the NCU in rural Rwanda's district hospital. The parents highlighted common experiences and difficulties adapting to the NCU environments both emotionally and physically, and financial barriers but also unique positive features of the NCU in Rwanda such as high levels of trust of parents for the healthcare providers, and uninterrupted access of mothers to their newborns.

The parents in our study shared similar experiences as reported in other previous studies in general – when their newborns were hospitalized, parents were understandably stressed and worried about their newborn's condition [7]. Consistent to other studies, inclusion in the provision of care contributed to parents' comfort, hope and confidence to keep caring for the newborns even after discharge [7,26,32]. According to other studies though, parents not only should be involved in the care, but also in the decision making about the newborn's care [33,34]. However, in our study, parents did not complain about not being involved in the decision-making for their newborns' care. On the contrary, many placed high trust in their healthcare providers. This could be due to many reasons. Parents may not feel sufficiently informed to make the decision, hence leaving it up to the healthcare providers to decide what is best for the newborn. The parents reported that they trusted the healthcare providers were providing the best possible treatment. There is a need for further research on the parental involvement in decision making in the NCU and other healthcare settings.

Having non-restrictive access to their newborns was one of the positive experiences for mothers. Such access, however, is not always allowed in many NCUs [23] and even other NCUs in Rwanda have restricted access [35]. Limited access to the NCUs for fathers and other family

members or caregivers was a source of concern for the parents in our study. Restricted NCU access is usually justified as necessary for infection control, however, studies present controversial evidence. One study in the United Kingdom found that restricted access was associated with a significant decline in respiratory infections [36], while another study in India showed that allowing parents in the NCU to directly participate in their newborn's care did not increase in hospital acquired infections [37]. NCU access restrictions have particularly been adopted during the COVID-19 pandemic where Neonatal Intensive Care Units that preserved the 24/7 parental presence decreased from 83 to 53% according to a global survey [38]. In all cases, education on hygiene practices are essential for all parents, visitors and providers. In the instances when the parents' full access to the NCU is scientifically justified as harmful to the newborn's health, an open and ongoing communication, emotional support, and the discussion on keeping the newborn connected to parents is needed [39].

Little involvement of fathers in newborn care has particularly been a source of negative experience for mothers and fathers. The lack of father involvement in the newborn care could reduce father's role to merely providing financial support. Studies have shown that little or no involvement of fathers in NCU caused the fathers to be scared of their preterm newborns, affected their early bonding, and eventually father-child relationships [3,40]. Recognizing the importance of parental access to newborns, some healthcare providers break the rules and allow occasional visitations – leading to some reports of "good nurses" and "bad nurses" [41]. Hospitals should reconsider the NCU visitation policy, and aim to deliver family-centered care to promote the best experience for the families and their newborns.

The quality of communication between caregivers and providers greatly impacted the experience of parents in the NCU. When NCU staff provided information about the newborn's

condition and showed a caring attitude, parents felt more hopeful, understanding, confident and a will to trust healthcare providers. Similar to the literature, our study highlighted that poor communication and blame by healthcare providers are a source of stress for parents [42].

The parental interactions and peer support were a source of positive experience for parents and should be encouraged. A systematic review has shown that informal or formalized peer support could improve the experiences and well-being of parents [43]. Such findings further reinforced the importance of clear communication from healthcare providers. When healthcare providers gave parents proper and clear orientation and instructions, such messages will be passed along among other parents.

Almost all respondents in our study mentioned the cost was a great source of stress. In Rwanda, 78.5% of the population were enrolled in the Community Based Health Insurance (CBHI) in 2019 [44]. Basic drugs and medical services are covered by CBHI. However, patients or their families are often required to pay for more specialized services and diagnostics. And if medication stock out occurs, they must purchase the medications for outside the hospitals. All these can contribute to extra financial burden. Additionally, indirect medical cost like transportation, food, and time from work are all imposing significant financial cost to the families. Similar to other studies, the cost of the NCU care, materials needed for the care, transportation, and food are serious challenges [45]. Studies to assess the cost related to NCU admission, and potential catastrophic out of pocket expenditures, should be investigated to inform policy makers if advocacy for expanding insurance coverage is needed.

Limitations

This study has some limitations. Most respondents in our study were women, and insights from fathers were relatively limited. The current study was conducted in a district hospital that receives support from a few non-government organizations. Not all Rwandan hospitals receive such support, thus the findings may not be generalizable to other Rwandan health facilities.

Conclusion

The admission of a newborn into the NCU is a source of stress for parents. Their stress is exacerbated by the environment including barriers to access by fathers and other family members, high-cost of neonatal care, and negative interactions with healthcare providers. However, the experience of care is improved by unrestricted access to the newborns by mothers as well as good communication with and trust of healthcare providers. Health facilities should also consider expanding the NCU access, encouraging peer support and actively involving parents in the care of their newborns. Further work is needed to understand the financial barriers for accessing NCU care.

Acknowledgements

The authors are grateful to Ruli District hospital administration and Neonatal Care Unit staff, for their support to allow this study to be conducted. We thank the parents who participated for taking the time to share their story.

List of abbreviations

- ABC: All Babies Count
- LMIC: Low- and Middle-Income Countries
- NCU: Neonatal Care Unit
- RNEC: Rwanda National Ethics Committee
- PTSD: Post-Traumatic Stress Disorder

Declarations

Ethics approval and consent to participate

The study was approved by the Rwanda National Ethics Committee (No. 107/RNEC/2019), University of Global Health Equity and Ruli District Hospital. Signed written consents were obtained from all participants.

Consent for publication

All co-authors read and approved the final manuscript.

Availability of data and materials

Not applicable

Competing interests

The authors declare that they have no competing interests.

Funding

The study was conducted with support from Grand Challenges Canada Saving Lives at Birth as part of the evaluation of the All Babies Count initiative. UGHE covered the cost of Dedoose software utilized in the analysis.

Authors' contributions

SB contributed to the study design, data collection, analysis, and writing the manuscript.

CK and RW contributed in the study design, data analysis and writing the manuscript.

JL contributed in writing the manuscript.

DK, SU, and JG contributed to the review of the manuscript.

References

1. WHO. Newborns: reducing mortality [Internet]. World Heal. Organ. 2018 [cited 2019 Dec 11]. p. 2017–20. Available from: https://www.who.int/news-room/fact-sheets/detail/newborns-reducing-mortality

2. Bhutta ZA, Das JK, Bahl R, Lawn JE, Salam RA, Paul VK, et al. Can available interventions end preventable deaths in mothers, newborn babies, and stillbirths, and at what cost? Lancet [Internet]. Elsevier; 2014;384:347–70. Available from: https://doi.org/10.1016/S0140-6736(14)60792-3

3. Adama EA, Sundin D, Bayes S. Ghanaian fathers' experiences of caring for preterm infants; a journey of exclusion. J Neonatal Nurs [Internet]. Elsevier Ltd; 2017;23:275–81. Available from: https://doi.org/10.1016/j.jnn.2017.05.003

4. EFCNI. Welcoming the family in the neonatal intensive care unit [Internet]. 2016. Available from: https://www.efcni.org/wp-content/uploads/2018/03/2016_05_02_Factsheet_A4_Welcoming_the_family_mE.pdf

5. Heidari H, Hasanpour M, Fooladi M. The experiences of parents with infants in Neonatal Intensive Care Unit. Iran J Nurs Midwifery Res [Internet]. Medknow Publications & Meida Pvt Ltd; 2013;18:208–13. Available from: https://www.ncbi.nlm.nih.gov/pubmed/23983756

6. Ncube RK, Barlow H, Mayers PM. A life uncertain – My baby's vulnerability: Mothers' lived experience of connection with their preterm infants in a Botswana neonatal intensive care unit. Curationis [Internet]. 2016 [cited 2019 Dec 6];39:9. Available from: http://www.curationis.org.za/index.php/curationis/article/view/1575

7. Obeidat HM, Bond EA, Callister LC. The Parental Experience of Having an Infant in the Newborn Intensive Care Unit. J Perinat Educ [Internet]. United States: Lamaze International;

2009;18:23–9. Available from:

http://www.ingentaconnect.com/content/lamaze/jpe/2009/00000018/00000003/art00006

8. Steyn E, Poggenpoel M, Myburgh C. Lived experiences of parents of premature babies in the intensive care unit in a private hospital in Johannesburg, South Africa. Curationis [Internet]. AOSIS Publishing; 2017 [cited 2019 Dec 6];40:1–8. Available from: http://www.curationis.org.za/index.php/curationis/article/view/1698

9. Dunn MS, Reilly MC, Johnston AM, Hoopes RD, Abraham MR. Development and Dissemination of Potentially Better Practices for the Provision of Family-Centered Care in Neonatology: The Family-Centered Care Map. Pediatrics [Internet]. 2006;118:S95 LP-S107. Available from: http://pediatrics.aappublications.org/content/118/Supplement_2/S95.abstract

10. American Academy of Pediatrics. Patient- and Family-Centered Care and the Pediatrician's Role. Pediatrics [Internet]. 2012;129:394 LP – 404. Available from: http://pediatrics.aappublications.org/content/129/2/394.abstract

11. Hagen IH, Iversen VC, Nesset E, Orner R, Svindseth MF. Parental satisfaction with neonatal intensive care units: a quantitative cross-sectional study. BMC Health Serv Res [Internet]. BioMed Central; 2019 [cited 2019 Dec 6];19:37. Available from: https://bmchealthservres.biomedcentral.com/articles/10.1186/s12913-018-3854-7

12. Bernard-Bonnin A-C. Maternal depression and child development. Paediatr Child Health [Internet]. Pulsus Group Inc; 2004 [cited 2018 Feb 24];9:575–83. Available from: https://academic.oup.com/pch/article/9/8/575/2648627

13. Guimarães H. The importance of parents in the neonatal intensive care units. J Pediatr Neonatal Individ Med [Internet]. 2015 [cited 2019 Dec 6];4:e040244. Available from: http://www.jpnim.com/index.php/jpnim/article/view/040244

14. Lefkowitz D, Baxt C, Evans J. Prevalence and Correlates of Posttraumatic Stress and

Postpartum Depression in Parents of Infants in the Neonatal Intensive Care Unit (NICU). J Clin Psychol Med Settings [Internet]. Boston: Springer US; 2010;17:230–7. Available from: https://www.ncbi.nlm.nih.gov/pubmed/20632076

15. Poehlmann J, Schwichtenberg AM, Bolt D, Dilworth-Bart J. Predictors of Depressive Symptom Trajectories in Mothers of Preterm or Low Birth Weight Infants. J Fam Psychol [Internet]. NIH Public Access; 2009 [cited 2018 Feb 24];23:690–704. Available from: http://www.ncbi.nlm.nih.gov/pubmed/19803605

16. Rogers CE, Kidokoro H, Wallendorf M, Inder TE. Identifying mothers of very preterm infants at-risk for postpartum depression and anxiety before discharge. J Perinatol [Internet]. 2013 [cited 2019 Dec 6];33:171–6. Available from: http://www.nature.com/articles/jp201275

17. Black MM, Walker SP, Fernald LCH, Andersen CT, Digirolamo AM, Lu C, et al. Advancing Early Childhood Development: from Science to Scale 1: Early childhood development coming of age: science through the life course [Internet]. Lancet (London, England). 2016. p. 77–90. Available from: https://www.openaire.eu/search/publication?articleId=od________267::06e6dfa66cc543657f622320235886d7

18. World Bank. World Bank Country and Lending Groups – World Bank Data 2019-2020 [Internet]. World Bank. 2019 [cited 2019 Dec 6]. p. 1–8. Available from: https://datahelpdesk.worldbank.org/knowledgebase/articles/906519-world-bank-country-and-lending-groups

19. Rwanda 2014-15. Stud Fam Plann [Internet]. John Wiley & Sons, Ltd (10.1111); 2016 [cited 2019 Dec 6];47:395–404. Available from: http://doi.wiley.com/10.1111/sifp.12002

20. Hansen A, Magge H, Labrecque M, Munyaneza RBM, Nahimana E, Nyishime M, et al. The development and implementation of a newborn medicine program in a resource-limited

setting. Public Heal action [Internet]. 2015;5:2220-8372:17. Available from: http://www.ncbi.nlm.nih.gov/pubmed/26400597%5Cnhttp://www.pubmedcentral.nih.gov/articlerender.fcgi?artid=PMC4525367

21. Charpak N, Tessier R, Ruiz JG, Hernandez JT, Uriza F, Villegas J, et al. Twenty-year Follow-up of Kangaroo Mother Care Versus Traditional Care. Pediatrics [Internet]. 2017 [cited 2019 Dec 6];139:e20162063. Available from: http://www.ncbi.nlm.nih.gov/pubmed/27965377

22. Karlsson V, Heinemann A-B, Sjörs G, Nykvist KH, Ågren J. Early Skin-to-Skin Care in Extremely Preterm Infants: Thermal Balance and Care Environment. J Pediatr [Internet]. 2012 [cited 2019 Dec 6];161:422–6. Available from: http://www.ncbi.nlm.nih.gov/pubmed/22497906

23. Musabirema P, Brysiewicz P, Chipps J. Curationis. [Internet]. Curationis. DENOSA; 2015 [cited 2018 Feb 24]. Available from: https://curationis.org.za/index.php/curationis/article/view/1499/1874#2

24. Tunçalp Ö, WM W, MacLennan C, OT O, AM G, Bahl R, et al. Quality of care for pregnant women and newborns—the WHO vision. BJOG An Int J Obstet Gynaecol [Internet]. Wiley/Blackwell (10.1111); 2015;122:1045–9. Available from: https://doi.org/10.1111/1471-0528.13451

25. Hawell E, Graham C. Parents ' experiences of neonatal care: A report on the findings from a national survey. Pick Inst Eur. 2011;1–2.

26. O'Brien K, Robson K, Bracht M, Cruz M, Lui K, Alvaro R, et al. Effectiveness of Family Integrated Care in neonatal intensive care units on infant and parent outcomes: a multicentre, multinational, cluster-randomised controlled trial. Lancet Child Adolesc Heal. 2018;2:245–54.

27. Rwanda Ministry of Health. Rwanda health management information system. 2017.

28. Rwanda Ministry of Health. Neonatology: Clinical Treatment Guidelines [Internet]. Kigali; 2012. Available from: www.moh.gov.rw

29. MOH Rwanda. Health Service Packages for Public Health Facilities • Rwanda Healthcare System • [Internet]. 2017. Available from: www.moh.gov.rw

30. Nyishime M, Borg R, Ingabire W, Hedt-Gauthier B, Nahimana E, Gupta N, et al. A retrospective study of neonatal case management and outcomes in rural Rwanda post implementation of a national neonatal care package for sick and small infants. BMC Pediatr [Internet]. BioMed Central; 2018 [cited 2020 Oct 15];18:353. Available from: https://bmcpediatr.biomedcentral.com/articles/10.1186/s12887-018-1334-1

31. Ruzibiza Y. 'They are a shame to the community … ' stigma, school attendance, solitude and resilience among pregnant teenagers and teenage mothers in Mahama refugee camp, Rwanda. Glob Public Health [Internet]. Routledge; 2020 [cited 2021 Feb 16]; Available from: https://doi.org/10.1080/17441692.2020.1751230

32. Ottosson C, Lantz B. Parental participation in neonatal care. J Neonatal Nurs. 2017;23:112–8.

33. Tandberg BS, Sandtrø HP, Vårdal M, Rønnestad A. Parents of preterm evaluation of stress and nursing support. J Neonatal Nurs. 2013;19:317–26.

34. Østergaard LR. Trust matters: A narrative literature review of the role of trust in health care systems in sub-Saharan Africa. Glob Public Health [Internet]. Taylor & Francis; 2015;10:1046–59. Available from: https://doi.org/10.1080/17441692.2015.1019538

35. Agaba F, Kalimba E, Khadka N, Mwali K, Shelus V, Wisner L. Situation Analysis of Inpatient Care of Newborns and Young Infants Authors : Matern. Child Surviv. Progr. Washington (DC); 2019.

36. Lakshmanan A, Szatkowski L, Zinna S, Clarkson M, McClaughry R, Soo S, et al. G109

Restricting visitors to the nicu significantly reduces nosocomial viral respiratory tract infections in babies. Arch Dis Child [Internet]. 2015;100:A47 LP-A47. Available from: http://adc.bmj.com/content/100/Suppl_3/A47.1.abstract

37. Verma A, Maria A, Pandey RM, Hans C, Verma A, Sherwani F. Family-centered care to complement care of sick newborns: A randomized controlled trial. Indian Pediatr [Internet]. 2017 [cited 2019 Dec 6];54:455–9. Available from: http://www.ncbi.nlm.nih.gov/pubmed/28667715

38. Darcy Mahoney A, White RD, Velasquez A, Barrett TS, Clark RH, Ahmad KA. Impact of restrictions on parental presence in neonatal intensive care units related to coronavirus disease 2019. J Perinatol [Internet]. Springer Nature; 2020 [cited 2021 Feb 19];40:36–46. Available from: https://www.nature.com/articles/s41372-020-0753-7

39. Murray PD, Swanson JR. Visitation restrictions: is it right and how do we support families in the NICU during COVID-19? J Perinatol [Internet]. Springer Nature; 2020 [cited 2021 Feb 19];40:1576–81. Available from: https://www.nature.com/articles/s41372-020-00781-1

40. Fisher D, Khashu M, Adama EA, Feeley N, Garfield CF, Ireland J, et al. Fathers in neonatal units: Improving infant health by supporting the baby-father bond and mother-father coparenting. J Neonatal Nurs [Internet]. Elsevier; 2018 [cited 2019 Dec 6];24:306–12. Available from: https://www.sciencedirect.com/science/article/abs/pii/S1355184118300930

41. Griffin T. A Family-Centered “Visitation” Policy in the Neonatal Intensive Care Unit That Welcomes Parents As Partners. J Perinat Neonatal Nurs [Internet]. 2013;27. Available from: https://journals.lww.com/jpnnjournal/Fulltext/2013/04000/A_Family_Centered__Visitation__Policy_in_the.12.aspx

42. Williams KG, Patel KT, Stausmire JM, Bridges C, Mathis MW, Barkin JL. The neonatal intensive care unit: Environmental stressors and supports. Int J Environ Res Public Health.

2018;15.

43. Altimier L. Newborn and Infant Nursing Reviews. Newborn Infant Nurs Rev [Internet]. W.B. Saunders; 2009 [cited 2019 Dec 6];9:129. Available from: https://www.sciencedirect.com/science/article/pii/S1527336909000841

44. Rwanda Social Security Board. Community-Based Health Insurance Fiscal Year 2018/2019 Annual Report. 2019.

45. Martinez AM, Khu DTK, Boo NY, Neou L, Saysanasongkham B, Partridge JC. Barriers to neonatal care in developing countries: Parents' and providers' perceptions. J Paediatr Child Health. 2012;48:852–8.

Supporting information

- S1 File. Semi-structured interview guide (English)
- S2 File. Semi-structured interview guide (Kinyarwanda)

Click here to access/download

Supporting Information - Compressed/ZIP File Archive

S_files_Semi_structured_interview_guide.zip

A ~~Qualitative~~ qualitative study to explore the experience of parents of newborns admitted to neonatal care unit in rural Rwanda

Formatted: Centered

Samuel Byiringiro[1,2*#a], Rex Wong[2,3], Jenae Logan[3,#b], Kaneza Deogratias[4], Joseph Gitera[5], Sharon Umutesi[6], Catherine M. Kirk[1,2,#c]

~~1.~~ [1] Maternal and Child Health Department, Partners In Health/Inshuti Mu Buzima, Rwinkwavu, Rwanda

~~2.~~ [2] Department, University of Global Health Equity, Butaro, Rwanda

~~3.~~ [3] Department, Yale University, New Haven, Connecticut, United States ~~of~~ America

~~4.~~ [4] Ruli District Hospital, Ministry of Health, Gakenke, Rwanda

~~5.~~ [5] ~~Department of Maternal and Child Health~~Clinical and Public Health Services Division, Ministry of Health, Kigali, Rwanda

~~6.~~ [6] ~~Department of~~ Maternal, ~~and~~ Child and Community Health Division, Rwanda Biomedical Center, Kigali, Rwanda

[#a]Current Address: Johns Hopkins University School of Nursing, Baltimore, Maryland, United States

[#b]Current Address: Partners In Health, Boston, MA

***Corresponding author~~:~~**

Email:~~Samuel Byiringiro~~ ~~(~~sam6as@yahoo.com (SB)~~)~~

~~These All authors contributed equally to this work.~~

Formatted: Indent: Left: 0.25", No bullets or numbering

Formatted: Font: (Default) Times New Roman, Not Superscript/ Subscript

Commented [CK1]: Rex to confirm

Formatted: Font: 12 pt

Formatted: Font: 12 pt

Formatted: Font: 12 pt

Commented [CK2]: Rex to confirm

Formatted: Highlight

Formatted: Font: (Default) Times New Roman, Not Superscript/ Subscript

Formatted: Font: (Default) Times New Roman, Not Superscript/ Subscript

Formatted: Font: (Default) Times New Roman, Not Superscript/ Subscript

Formatted: Font: (Default) Times New Roman, Not Superscript/ Subscript

Formatted: Font: (Default) Times New Roman, Not Superscript/ Subscript

Formatted: Font: (Default) Times New Roman, Not Superscript/ Subscript

Formatted: Font: (Default) Times New Roman, Not Superscript/ Subscript

Formatted: Font: (Default) Times New Roman, Superscript

Formatted: Font: (Default) Times New Roman, Superscript

Formatted: Indent: Left: 0.5"

Formatted: Font:

Field Code Changed

Formatted: Font: (Default) Times New Roman, Font color: Auto

Formatted: Font: (Default) Times New Roman, Font color: Auto

Formatted: Font: (Default) Times New Roman, Font color: Auto

Formatted: Font: Times New Roman

Abstract

Background: Neonatal Care Units (NCUs) provide special care to sick and small newborns and help reduce neonatal mortality. For parents, having a hospitalized newborn can be a traumatic experience. In sub-Saharan Africa, there is limited literature about the parents' experience in NCUs.

Objective: Our study aimed to explore the experience of parents in the NCU of a rural district hospital in Rwanda.

Methods: A qualitative study was conducted with parents whose newborns were hospitalized in the Ruli District Hospital NCU from September 2018 to January 2019. ~~In-depth I~~interviews were conducted using a semi-structured guide in the participants homes by trained data collectors. Data were transcribed, translated, and then coded using a structured code book. All data were organized using Dedoose software for analysis.

Results: Twenty-one interviews were conducted primarily with mothers (90.5%, n=19) among newborns who were most often discharged home alive (90.5%, n=19). Four themes emerged from the interviews. These were the parental adaptation to having a sick neonate in NCU, adaptation to the NCU environment, interaction with people (healthcare providers and fellow parents) in the NCU, and financial stressors.

Conclusion: The admission of a newborn to the NCU is a source of stress for parents and caregivers in rural Rwanda, however, there were several positive aspects which helped mothers adapt to the NCU. The experience in the NCU can be improved when healthcare providers communicate and explain the newborn's status to the parents and actively involve them in the care of their newborn. Expanding the NCU access for families, encouraging peer support, and

Formatted: Font: Bold

ensuring financial accessibility for neonatal care services could contribute to improved experiences for parents and families in general.

Keywords: prematurity, neonatology, family-centered care, neonatal care unit, nurses, patient satisfaction, patient experience

Background

Formatted: Font: Bold

Neonatal mortality makes up nearly half of all deaths among children under five years of age [1]. It is estimated that nearly 1.9 million newborn deaths can be averted by 2025 if quality maternal and newborn care interventions were implemented [2]. Expansion of access to specialized neonatal care units (NCUs) for sick and small newborns is one of the global priorities to reduce neonatal mortality [1]. Despite the tremendous benefits of NCUs in achieving better neonatal health outcomes, studies exploring experience of parents in NCUs have shown it to be a stressful experience for families [3–8].

While there is a large body of literature regarding parental experience in NCUs in western countries, very little is known in Sub-Saharan Africa [9,10]. A small number of studies in South Africa showed that parents often experience negative emotional feelings and various challenges in NCUs [8,11]. Many parents expressed depressive symptoms and inability to bond with their newborns [12–16]. Some newborns were abandoned in the NCU, and this disruption of parental care can contribute to inferior early child development outcomes, lower educational attainment, and poorer lifetime economic earnings [17]. However the findings and recommendations from South Africa - a high middle-income country, are difficult to transfer to other Sub-Saharan African nations - predominantly low income countries [18].

Between 2005 and 2015 , Rwanda has documented a rapid decline in under-five child mortality from 152 to 50 deaths per 1,000 live births and neonatal mortality from 37 to 20 deaths per 1,000 live births [19]. Despite such tremendous progress, neonatal deaths still contribute to 40% of all under-five deaths in Rwanda [19]. In order to accelerate the reduction in neonatal mortality, the Rwandan Ministry of Health launched the National Neonatal Care Protocol in 2012 addressing the gaps directly related to clinical neonatal care [20] and scaled-up hospital

level neonatal care countrywide. However, other than for skin to skin care, the protocol does not integrate parents into the NCU environment [21,22]. One quantitative study found high stress score and the predominant stressors were the babies' appearance and behavior among parents of newborns admitted to NCU in an urban tertiary hospital [23].

Quality healthcare requires both the provision of evidence-based clinical care to newborns and also ensuring a positive experience of the newborn and their families [24]. High quality care cannot be fully achieved unless the experience of care meets the needs of families, which requires knowing their experiences, and designing appropriate interventions [24–26]. Accordingly, this study explored the experience of parents whose neonates were hospitalized and discharged from the NCU in a district hospital in rural Rwanda. The results of this study can add to the existing literature on the experience of parents with newborn children in the NCU in low- and middle-income countries in the sub-Saharan Africa region.

Methods

Formatted: Font: Bold

Setting

This study was conducted in the catchment area of Ruli District Hospital – a rural public hospital located in Rwanda's Northern Province. With 10 beds and 5 incubators, Ruli District Hospital's NCU also serves as a referral site for hydrocephalus surgical treatment. In 2017, there were 459 NCU admissions [27]. From 2017-2019, Ruli District Hospital received support to improve the quality of care for newborns through the All Babies Count (ABC) program implemented by Partners In Health/Inshuti Mu Buzima and the Ministry of Health.

NCUs at District hospital levels in Rwanda

Formatted: Font: (Default) Times New Roman, Bold, Font color: Auto

NCUs at the district level in Rwanda provide preventive, curative, and rehabilitation care to the small and sick newborns, and those at risk for complications [28,29]. The majority of babies who are referred to the district level NCUs are usually those born too early and/or too small (with weight below 2.5 kg), but occasionally too big (above 4 kg); babies with birth asphyxia; and babies with risk for infection as stated in the national clinical guideline (including but not limited to babies born at home, from mothers who presented with a fever during labor or 24 hours after delivery, or rupture of membranes 18 hours before delivery) [28,30].

District level NCUs exist as a separate unit from maternity even though they work closely together. They treat newborns from both the health centers ~~pf~~ of the hospitals' catchment area and the hospital maternity. Babies born at home are automatically referred to the NCU for risk of infection, and while those born at the health centers and the hospital maternities are evaluated for any necessity to refer to them to the NCU. If transferred to the NCU, hospitals' procedures vary but at Ruli DH, the baby's mother or closest relative accompany the baby inside the NCU ~~.~~[28].

NCUs in Rwanda provide care at varying capacities yet the health service package for public health facilities determines that they should be able to offer supportive oxygen to babies with birth asphyxia, continuous positive airway pressure to premature and term babies with respiratory distress, antibiotic therapy, thermal regulation support, fluid and electrolytes when necessary to name a few [29]. Specialized neonatal care is usually absent at district levels and newborns who require it are sent to referral hospitals' NCUs.

Design

We used phenomenological qualitative descriptive and contextual approach in this qualitative study of parent's lived experiences [31]. Semi- structured interviews were conducted to understand the experiences of parents having a newborn hospitalized and discharged from the NCU.

Study Participants

Parents or primary caregivers of neonates discharged from the NCU between September 2018 and January 2019 (three months prior to data collection) were included in the study. They had to be at least 18 years of ageTo ensure that parents and caregivers had been amply exposed to the NCU experience, we only included those and with newbornswhose newborns were admitted to the NCU for at least three days. While some mothers and caregivers were aged below 18 years, we excluded them since their experience tends to be unique because of stigma associated with teenage pregnancy in Rwanda [31]. The acquisition of consent for the participants aged below 18 is additionally different. To ensure diversity and a mix of experiences, wWe purposively stratified the samples by distance (living within and more than 1-hour walking distance from the facility), by admitting diagnosis (prematurity and other reasons), and outcome (died or alive at discharge).

Recruitment procedure

The recruitment took place between December 2018 and January 2019. After acquiring the approval to access data from Ruli District Hospital leadership, we made the list of eligible potential participants and their geographical locations from the NCU registry. We contacted the

Formatted: Font: (Default) Times New Roman, Bold

Formatted: Font: (Default) Times New Roman, Bold

Formatted: Font: (Default) Times New Roman, Bold

Formatted: Normal

Formatted: Font: (Default) Times New Roman

nearby Community Health Worker (CHW) to request permission on our behalf and book us an appointment to the potential participants' homes. On the appointment date, the interviewer was accompanied by the CHW to the potential participant's home which is where the data collection took place.

Data collection tool and procedures

Formatted: Font: (Default) Times New Roman, Bold

Formatted: Font: (Default) Times New Roman, Bold

Interviews were conducted using a semi-structured interview guide that included questions related to the parents' and caregivers' feelings on neonatal admission and their experience in the NCU, the quality of communication with healthcare providers, their involvement in the newborn's care, and the support received from the hospital staff, relatives, or others during the stay. We developed the The interview guide in English was translatedand translated to it to Kinyarwanda - the local language used for conducting the interviews. .

The interview guide was pre-tested and piloted on two parents before the actual data collection. The pilot did not lead to changes in the protocol hence its participants were included in the analysis. Participants who fulfilled the selection criteria were identified via the NCU register and were contacted through their respective Community Health Workers and were invited to participate in the interviews. Interviews were conducted at the participants' homes on the appointment dates until saturation was reached. Two female data collectors who were not healthcare providers nor worked in the study district were trained to conduct the interviews. Husbands and wives were interviewed separately. The interviewer provided information about the study to the participants and clarified that participation was voluntary. Those who accepted to take part in the study provided wWritten informed consents were obtained before the interview beginss. The interviews took place the quiet place at the participants homes. The study

participants received a gift consisting of a baby hat and a bar of soap. The Principal Investigator listened to the interviews at the end of each data collection day to determine if the saturation was reached..

Data ~~Analysis~~analysis

The iInterviews were transcribed and translated, then coded by two independent investigators. We explored the literature on the experience of parents in typical low-income settings' NCUs and used it to create the preliminary codebook. The codebook was amended using pilot interviews. The independent coders met with the Principal Investigator to resolve any discrepancies by discussion and revision of the definition of the codes. ~~The codebook was created based on the literature, and the pilot interviews.~~ The transcripts were coded then grouped into themes. The data analysis was conducted using Dedoose Software.

Formatted: Font: (Default) Times New Roman, Bold

Formatted: Font: (Default) Times New Roman, Bold

Ethics

Formatted: Font: (Default) Times New Roman, Bold

The study was approved by the Rwanda National Ethics Committee (No. 107/RNEC/2019) and the Institutional Review Board for the University of Global Health Equity. Ruli District Hospital provided approval for NCU data access and participants signed informed consent prior to the beginning of the interviews. To protect the participants' confidentiality, we utilized participant numbers in the transcripts instead of their names, and we de-identified all quotes included in the results by removing details that could lead to the recognition of the individual participant or their healthcare provider..

Results

Formatted: Font: (Default) Times New Roman, 18 pt, Bold

A total of 21 interviews were conducted (Table 1). Participants were aged between 20 and 56. Nineteen (90.5%) were female participants. Seventeen (80.9%) of the participants were married and 4 (19.0%) were single. Nineteen (90.5%) had children who were alive, while 2 (9.5%) died prior to discharge from NCU. Eight (31.0%) lived within an hour walking distance to the hospital while 13 (61.9%) had to either take the bus or a motorcycle or walk for more than an hour to the hospital from their homes. Prematurity (42.8%) and infection risk or fever (14.2%) were the most common reasons for admission. All participants had health insurance.

Four overarching themes were generated from the interviews with parents regarding their experience of having their newborn hospitalized in NCU at Ruli DH. Theme one consists of parental emotional adaptation to the newborn care in the NCU. The second theme describes the parental adaptation to the NCU environment which includes the physical environment and rules and regulations of the NCU. The third theme covers the experience of parents with other people in the NCU and the last theme discusses the Financial aspects of the care and support received at Ruli DH.~~H~~

Formatted: Font: (Default) Times New Roman, Font color: Auto

Formatted: Font: (Default) Times New Roman, Font color: Auto

Formatted: Font: (Default) Times New Roman, Font color: Auto

Table 1. Demographic characteristics of participants

**Characteristics**		**N (%)**
Total Participants		21
Age (n=19)	20-29	8 (38%)
	30-39	8 (38%)
	>=40	3 (15%)
	Undeclared	2 (9%)
Gender	Male	2 (9.5%)
	Female	19 (90.5%)
Newborn's outcome at discharge	Alive	19 (90.5%)
	Dead	2 (9.5%)
Marital status	Married	17 (80.9%)
	Single	4 (19.0%)
Distance to hospital	<1 hour	8 (38.0%)

	>1 hour	13 (61.9%)
Reason for admission	Prematurity	9 (42.8%)
	Low Birth Weight	2 (9.5%)
	Infection risk/Fever	3 (14.2%)
	Other*	7 (33.3%)
Health Insurance	Yes	21 (100%)

[a]Other: asphyxia, congenital malformation, jaundice, not sucking on breast, respiratory distress, cord bleeding

Theme 1. Parents' Emotional Adaptation to the newborn care in the NCU

Parents' emotional adaptation to receiving newborn care in the NCU was first characterized by a mix of negative feelings and emotions resulting from the quick roll out of events in the short time from stress of delivery to the admission in the NCU. The parents found the NCU environment to look strangely different because of the unusual equipment and the type of care newborns receive. They felt for and feared that the baby was being hurt by the different nursing and medical care and procedures.

1.1. Stress due to baby's condition ~~shared fear and emotions while they were adapting to an unfamiliar care environment in the NCU. They shared challenges with the cold temperature of the NCU, but also reported the NCU to be clean and calming.~~

Many parents reported feeling fearful when they were informed that their newborn required admission to the NCU. They feared that ~~their~~ the ~~children~~ child ~~may~~ could die and that caused them sadness, uneasiness, hopelessness, disturbance, and anxiety.

Formatted: Line spacing: single

Formatted: Space After: 10 pt, Line spacing: single, Keep with next

Formatted: Font: (Default) Times New Roman, Bold

Formatted: Font: (Default) Times New Roman, Font color: Auto

Formatted: Font: (Default) Times New Roman, Font color: Auto

Formatted: Font: (Default) Times New Roman, Font color: Auto

Formatted: Font: (Default) Times New Roman, Font color: Auto

Formatted: Font: (Default) Times New Roman, Font color: Auto

Formatted: Font: (Default) Times New Roman, Font color: Auto

Formatted: Font: (Default) Times New Roman, Font color: Auto

Formatted: Font: (Default) Times New Roman, Font color: Auto

Formatted: Font: (Default) Times New Roman, Font color: Auto

Formatted: Font: (Default) Times New Roman, Font color: Auto

"There was no other way I could understand it... I didn't even feel hopeful because the baby was too premature. I was just waiting for what God was to give me. My baby only weighed 1.2 kg. I felt hopeless and fe~~e~~lt the healthcare providers were struggling for nothing." (Mother, age 36).

Several mothers reported a very emotional response to their newborn's admission:

"I felt very sad and I am telling you that I would suddenly burst into tears. I was alone with the baby always expecting to be discharged the following day. I felt disturbed, wept repeatedly, then breastfeed the baby but suddenly find myself crying." (Mother, age 28).

"I cried. Other mothers used to say that no other person enters in there [NCU]. I felt like it was over for me when I saw myself referred there. I thought that I would not leave soon and I imagined that my baby would eventually die. ~~The place [NCU] is strange. Before this time, we used to bring food to mothers whose babies would be hospitalized in the NCU. We would not get in contact with them [other mothers]. We would see them from a distance, and you could not see their babies. When I realized that it was my turn to go there,~~ I felt very sad, and it was hard for me to accept the situation" (Mother, age 25).

1.2. Stress due to the NCU outlook and the visual sight of the baby

Many parents reported negative feelings caused by the look at the different tubes and wires attached to the newborns inside the incubators, the sound of the equipment and sometimes the care provided to the newborns. While the fathers were not allowed into the incubator room part of the NCU, one father reported managing to trespass into that room and described how he felt when he saw his fifth newborn in the incubator for prematurity:

"I felt scared to see the machines. You say, look at these machines, and the baby is lying in them naked. I was not sure that my baby would survive." (Father, age unknown).

Formatted: Font: (Default) Times New Roman, Font color: Auto

Formatted: Font: Not Italic

Formatted: Font: (Default) Times New Roman, Font color: Auto

Formatted: Indent: Left: 0"

Formatted: Font: (Default) Times New Roman, Font color: Auto

Another mother shared:

> *"I was stressed because I was thinking that she (my baby) would not survive. I saw that my baby was on oxygen, and another noisy machine, I lost hope because the baby spent the whole week in the incubator. When I arrived at the hospital, I was so worried that my baby would not make it."* (Mother, age 35).

1.3. Stress due to that fear the baby is being hurt during care

Formatted: Font: (Default) Times New Roman, Not Italic, Font color: Auto

Formatted: Indent: Left: 0"

Additionally, parents feared that their newborn may be hurt by the care provided in the NCU. The fear was particularly higher when the parents thought the provider was not experienced enough (student for instance) to care for the sick baby.

Formatted: Font: (Default) Times New Roman, Font color: Auto

> *"He [a nursing student] held it [a needle] and pierced without first looking for where the vein was, and he attempted three times. The third time, I told him to stop paining my baby."* (Mother, 32).

Theme 2. Parental Adaptation to the NCU Environment

Formatted: Font: (Default) Times New Roman, Bold

Formatted: Font: (Default) Times New Roman, Bold

~~Caregivers~~ Parents reported some regulations that were positive – such as uninterrupted access to their newborn but other regulations of no visitors, no caregivers, and no eating inside the ward stressed mothers. The procedures of always cleaning hands, changing shoes, and putting on different clothing (aprons) are a source of stress to some mothers even though some mothers appreciated them due to the resulting calmness in NCU. Caregiver's stress was worsened by insufficient induction to the NCU contributing to a lack of understanding of NCU rules and procedures ~~and poor adaptation to the environment~~.

Formatted: Font: (Default) Times New Roman, Font color: Auto

Formatted: Font: (Default) Times New Roman, Font color: Auto

Formatted: Font: (Default) Times New Roman, Font color: Auto

Formatted: Font: (Default) Times New Roman, Font color: Auto

Formatted: Font: (Default) Times New Roman, Font color: Auto

2.1. Being part of the baby's care

Mothers reported that having full access to the newborn any time throughout hospitalization, even when the newborns were inside incubators or on phototherapy, brought them comfort. Having some responsibility for the care of their newborns, ranging from paying the bills, to bringing required materials, to feeding or bathing their newborn, improved their feelings about the situation even though the part of responsibility was occasionally a source of stress to parents who were unable to bring fulfill all their responsibilities (Paying bills, providing newborn materials such as clothes, bringing hygiene materials and so on)~~:~~.

> *"~~Of course, we, as mothers, feel very stressed. However, t~~The healthcare providers told us, ~~there was no problem,~~ we could go at any time to have a look at our babies. They told us to be close to our babies. We hence used to come look at our babies and [go] back whenever."* (mother, age 35).

> *"They [nurses] told me to go and breastfeed my baby anytime I wanted. I felt no problem because I was free to go and see the baby anytime."* (mother, age 22).

While mothers ~~could~~ rejoiced in being able to access their newborns at any time~~,~~, ~~other~~ the ~~mothers~~adaptation and stay was deranged when mothers ~~were stressed~~ were ~~by not being~~ unable to care for their newborns such as lacking breast milk. The stay was additionally unpleasant for some mothers~~,~~ who ~~or provide sufficient breastmilk. They also~~ lacked ~~ed~~ means to follow up ~~on~~ their own health because they were ~~usually~~ still sick when the newborns were transferred to the NCUs.

> *"What stressed me is when they told me to express breastmilk for my baby. The baby stayed in the machines on oxygen, and when it was time to start pumping breast milk for the baby, I could not get it."* (mother, age 36).

Formatted: Font: (Default) Times New Roman, Font color: Auto

Formatted: Font: (Default) Times New Roman, Font color: Auto

Formatted: Font: (Default) Times New Roman, Font color: Auto

Formatted: Font: (Default) Times New Roman, Font color: Auto

Formatted: Font: (Default) Times New Roman, Font color: Auto

Formatted: Font: (Default) Times New Roman, Font color: Auto

Formatted: Font: (Default) Times New Roman, Font color: Auto

Formatted: Font: (Default) Times New Roman, Font color: Auto

Formatted: Font: (Default) Times New Roman, Font color: Auto

Formatted: Font: (Default) Times New Roman, Font color: Auto

Formatted: Font: (Default) Times New Roman, Font color: Auto

Formatted: Font: (Default) Times New Roman, Font color: Auto

"Because of taking care of the baby while I was still sick, I became sicker and felt as if I was dead. I did not know what I was sick of but when I went to seek care I had high fever and was shivering." (mother, age 33).

2.2. Access to their babies

The fathers were the only caregivers other than the mothers reported to have some access into the general room (Kangaroo Mother Care Unit), though they had limited visiting hours and were not allowed to touch or hold the newborns, or see them if they were in the incubators' room. Other visitors were not allowed into the NCU. The mothers felt very sad when the newborn passed away without having the father see them.Mothers expressed the challenges and stress related to not having their husbands be able to see their newborn, especially if they had no hope that the newborn would survive.

A mother reported her feeling that the father did not see their newborn before she died:

"I told one of the other mothers in the NCU that I was very sad because my baby was going to die before her father could at least see her. The mother told me that I should have requested it before, and I told her that nobody advised me to ask a healthcare provider for the permission so that my husband could visit my baby." (mother, age 35)

2.3. Orientation to the NCU

Parents did not appreciate the fact that they were not provided a rigorous initial orientation so that they can consciously abide by on the NCU regulations. They would find themselves frustrated from by the things about which they should have been informed about at admission. Most of quarrels between parents and healthcare providers resulted from the tendency of mothers to bring visitors to the NCU.

Formatted: Font: Not Italic

Formatted: Indent: Left: 0"

Formatted: Font: (Default) Times New Roman, Font color: Auto

Formatted: Font: (Default) Times New Roman, Font color: Auto

Formatted: Font: (Default) Times New Roman, Font color: Auto

Formatted: Font: (Default) Times New Roman, Font color: Auto

Formatted: Font: (Default) Times New Roman, Font color: Auto

Formatted: Font: (Default) Times New Roman, Font color: Auto

Formatted: Font: (Default) Times New Roman, Font color: Auto

Formatted: Font: (Default) Times New Roman, Font color: Auto

Formatted: Font: (Default) Times New Roman, Font color: Auto

Formatted: Font: (Default) Times New Roman, Font color: Auto

Formatted: Font: (Default) Times New Roman, Font color: Auto

"She [healthcare provider] asked me why I had violated the rules [allowing the father of the baby to visit]. ~~I responded that I had read that fathers are allowed to come and see the baby.~~ She replied that I should not allow him in without telling her first. I told her that I had read the regulation but did not know that I should get approval from her before bringing him inside." (Mother, age unknown).

"They [healthcare providers] should explain to the mother where they were taking the baby and explain the life there. For instance, after I arrived, nobody explained the rules and regulations of the place except reading it for myself. None told me the procedure of things in the unit. They should improve that.~~, There are people who made mistakes because they were not told before.~~" (Mother, age 25).

Formatted: Font: (Default) Times New Roman, Font color: Auto

2.4. Physical environment of NCU

Formatted: Space Before: 6 pt, Line spacing: Double

In addition to the stressors from the NCU regulations, ~~parents~~ mothers reported the NCU interior to have low temperature which make it chilly ~~be cold~~. They even feared that the coldness they felt affected the babies as well. Additionally, the nature of the NCU care makes mothers unable to rest as many of them had to watch over their newborns day and night.

Formatted: Font: (Default) Times New Roman, Font color: Auto

Formatted: Font: (Default) Times New Roman, Font color: Auto

Formatted: Font: (Default) Times New Roman, Font color: Auto

Formatted: Font: (Default) Times New Roman, Font color: Auto

Formatted: Font: (Default) Times New Roman, Font color: Auto

"Cold... ohhhh, it was too cold. Our babies were freezing and were losing weight. The room was freezing." (mother, age 44).

Despite the many stressful aspects of the NCU, ~~other~~ mothers reported the environment to be safe from disorder and robbery, clean and quiet which provides a sense of comfort and positive feelings. Some mothers reported appreciating the safeguard put in place to minimize visitations explaining that such measures allow the NCU environment to remain clean, and silent which they thought is good for the sick newborns.

Formatted: Font color: Auto

Formatted: Font color: Auto

"I thought the place would be smelling bad. I, however, found it different. They [healthcare providers] teach them [mothers] morning and evening to have hygiene so that even newcomers can be informed about hygiene." (mother, age 43).

"I was very happy with the hygiene of the NCU. I was happy with the way they restrict the access into the Unit where there are babies born with problems. You see, allowing many people inside would cause the room of sick babies to be stuffy. They avoided that. The way they prohibit the entry of shoes from outside is good." (mother, age 27)

"You enter and you can even make a prayer silently in your mind to avoid making noise. You make sure there is no noise in the NCU, no one is allowed to scream, the people inside are so quiet. It is not authorized to make phone calls or other devices that may produce noise." (mother, 35).

Theme 3. Experiences with ~~Other~~ other ~~People~~ people in NCU

~~Caregivers~~ Parents reported having both positive and negative interactions with healthcare providers, and an overall strong sense of trust for healthcare providers. Other mothers in the NCU were reported to be a consistent source of support (emotional and material) for each other.

3.1. Interactions with healthcare providers

A mix of healthcare provider attitudes, some positive and others negative, were reported by parents. They were appreciative of the healthcare providers who explained the care their newborns received, provided guidance and updates on the newborn's condition, and encouraged them to ask questions.

Formatted: Font: (Default) Times New Roman, Font color: Auto

Formatted: Font: (Default) Times New Roman, Bold

Formatted: Font: (Default) Times New Roman, Bold

Formatted: Font: (Default) Times New Roman, Bold

Formatted: Font: (Default) Times New Roman, Font color: Auto

Formatted: Font: (Default) Times New Roman, Font color: Auto

Formatted: Font: (Default) Times New Roman, Font color: Auto

Formatted: Font: (Default) Times New Roman, Font color: Auto

Formatted: Font: (Default) Times New Roman, Font color: Auto

"There was one woman [healthcare provider] who used to talk to us. She would say, 'Do you have any questions?', and we often responded, 'no'. She would then ask us what she would have taught us. We were more comfortable with that healthcare provider." (Mother, age 28).

"We would joke with healthcare providers and occasionally forget that our babies are hospitalized. They would come and comfort us telling us that our babies would get cured. You could notice that they were doing whatever possible to converse with us." (Mother, age 33)

The provision of information about the findings of assessments contributed to the parent's understanding of the newborn's condition and was one key factor to the positive feelings and comfort to the mothers in the otherwise hopeless conditions.

"It was my first time to see such ~~kind of~~a disease [jaundice], my first time to hear of it. I had~~ a~~ hoped that he ~~will~~ would get well soon. After they [healthcare providers] explained the condition to me, I felt ensured that the child shall get cured and I had no problem~~. In fact, because the healthcare provider explained the baby's condition to me, I did not ask again. However, before explaining to me, I was very curious to know what caused the disease but they gave me explanations~~." (Mother, age 22).

"When I arrived, they [healthcare providers] examined the baby, looked at her vomiting and the problem in her eyes and they comforted me saying it would go away little by little. They advised me how to position my baby while breastfeeding and it decreased gradually. Gradually as they talked to me and comforted me, I regained the mood." (Mother, age 28).

Formatted: Font: (Default) Times New Roman, Font color: Auto

Formatted: Font: (Default) Times New Roman, Font color: Auto

Formatted: Font: (Default) Times New Roman, Font color: Auto

However, some healthcare providers were reported to have a bad attitude which made the NCU stay stressful and challenging for some mothers. Parents reported a negative experience of interacting with some healthcare providers who spoke in a rude tone, provided no guidance or information, and showed no empathy while requesting newborn care materials from parents.

Formatted: Font: (Default) Times New Roman, Font color: Auto

> *"Whenever healthcare providers entered, I trembled ~~felt fear~~ because they entered speaking roughly, except that you ought to get used to it. Once, ~~when the baby was inside a machine, they wore only pampers because they were naked inside the machines.~~ I went outside immediately after breastfeeding. A few minutes after, when I came back the baby had passed stools. The healthcare provider immediately talked to me in a rude voice. I tried to explain that I was there with my baby a few seconds ~~ago~~before. She refused to understand and roughly said I should look after my baby at every moment."* (Mother, age 22).

Formatted: Font: (Default) Times New Roman, Font color: Auto

Formatted: Font: (Default) Times New Roman, Font color: Auto

> *"They yelled at me asking me why I had not yet bought clothes for my baby. I begged and told them that I could not afford to buy the clothes because I am poor. She rudely replied that poor people do not go to seek care at Ruli hospital."* (Mother, age 36).

Blaming parents for the newborn's condition was another attitude repeatedly reported by parents. In most cases, the parents reported that healthcare providers blamed them for not taking good care of their babies or not fulfilling their responsibilities (such as providing needed materials for newborn care) hence leading to worsening of the condition.

Formatted: Font: (Default) Times New Roman, Font color: Auto

> *"My baby had a fever and was vomiting. I called the healthcare provider who was on day duty and she said that it was because of me that I was not holding my baby well."* (Mother, age 22).

"They [healthcare providers] kept telling me that I had refused to go to seek care in Kigali when I was referred. I told them that I had no means." (Mother, age 36).

Most of the parents interviewed expressed high levels of trust in healthcare providers, including those whose newborns died prior to discharge. Parents reported that they trusted that providers were doing their best to save the newborns.

"I wished to be involved, but I did not decline any treatment because I could see them coming to treat my baby and I felt that because they are healthcare providers my baby would be cured." (Mother, age 33).

"I felt that because my baby is in the hands of healthcare providers, she would be fine. They would comfort us saying that no baby would die anymore, and we felt hopeful. After that, they took an exam and the result was good and I was very happy that my baby was fine. They took care of my baby. They tried whatever possible." (Mother, age 25).

3.1. Interactions with other parents

In addition to turning to their partners, family, and relatives for support at the time of admission and during the stay, fellow mothers in NCU provided emotional and material (food, clothes, and other consumables) support which was a great comfort for many.

"Other mothers are the ones who helped me feel better. When I looked at their babies and they told me that their babies were sicker when they arrived than [at] that time, I felt better." (mother, age 27).

"There was another woman next to me, who had also given birth to twins and whose caregiver was her mother. They used to lend me materials and because she also came from far, when they would have brought them food they cooked and shared with me. I did

Formatted: Indent: Left: 0", Line spacing: single

Formatted: Font: (Default) Times New Roman, Font color: Auto

Formatted: Font: (Default) Times New Roman, Font color: Auto

not have people who could bring me food, so, I survived because there were people of goodwill at the hospital." (mother, age 25).

Other mothers also helped explain the situation of the NCU to others when healthcare providers did not:

> *"We had to transmit the information to the mothers who were new and did not know these instructions because healthcare providers were not always in [a] good mood of communicating."* (Mother, age 25).

> *"There were mothers without caregivers. They go into the eating hall to see if someone can give them food. We do share food with them because one cannot finish it. I have noticed that people help each other., ~~because of the service that takes place at the hospital. When you see how healthcare providers try to support us, and you wonder how we cannot support each other's mothers as well.~~"* (Mother, age 28).

Formatted: Font: (Default) Times New Roman, Font color: Auto

Theme 4. Financial aspects of the care in NCU and support received

Formatted: Font: (Default) Times New Roman, Bold

Many mothers and fathers ~~said~~ reported that the financial burden due to NCU admission was challenging and was a stress for them. They had difficulty in paying many of the NCU requirements such as specific clothing, basins, flasks as well as the hospital fee. Despite all participants having health insurance, many could not afford the co-payment. Two interviewees reported they had to sell a portion of their land to pay for the NCU costs.

Formatted: Font: (Default) Times New Roman, Font color: Auto

Formatted: Font: (Default) Times New Roman, Font color: Auto

> *"I was wondering how I would get money to pay the invoice that was 99 times more than my salary. People told me that I must sell the land to pay medical invoices. I was scared. I was wondering how I will survive when I go home because I had already sold my land in*

order to buy clothes [for the baby]. I was speechless and there were times when I did not have breast milk because of problems." (mother, age 44).

"I was stressed because of the 30,000 RWF medical bill for spending a week and three days in the hospital. I was wondering where I would get the money if I had to spend one more week in the hospital. I was confused but I was also ready to sell my house to be able to pay the medical bill." (Mother, age 34). She eventually sold her land to pay the bills.

Even sometimes when the NCU was not as expensive as expected, the thought of the potential cost caused a lot of stress.

"When we went to the hospital, we wondered how we could pay the bill. ~~We ask ourselves so many questions.~~ I was afraid that I would not get enough money to pay the medical bills and would be held in custody at the hospital. But, luckily, I was not charged a lot of money because I had health insurance. I did not have to sell any of my goods." (mother, age 35).

The cost of hospitalization was a major source of stress, and any forms of intermittent support they had received from others, like porridge and food received from the Catholic sisters or university students supporting the hospital were very much appreciated by the parents.

"Every morning they [healthcare providers] bring the porridge and bread. I cannot blame them for anything. The healthcare providers bring the hungry people food from the Sisters. They do whatever possible." (mother, age 36).

"The most important thing that they helped us with was the free porridge in the morning. It was very impactful because a mother cannot breastfeed the baby without eating the porridge. It is the best help they gave us. We ate the porridge happily with the peace of mind." (mother, age 25).

Discussion

Parents shared both positive and negative aspects of their experience ~~at~~ inside the NCU in rural Rwanda's district hospital~~,~~. The parents highlighted ~~ting~~ common experiences ~~seen among other parents such as stress, sadness~~and difficulties adapting to the NCU environments both emotionally and physically,~~,~~ and financial barriers but also unique positive features of the NCU in Rwanda ~~that were a positive~~ such as high levels of trust of parents for the healthcare providers, and uninterrupted access of mothers to their newborns.

The parents in our study shared similar experiences as reported in other previous studies in general – ~~:~~ when their newborns were hospitalized, parents were understandably stressed and worried about their newborn's condition [7]. Consistent to other studies, inclusion in the provision of care ~~could~~ contributed to parents' comfort, hope and confidence to keep caring for the newborns even after discharge [7,26,32]. According to other studies though, parents not only should be involved in the care, but also in the decision making about the newborn's care [33,34]. However, in our study, parents did not complain about not being involved in the decision-making for their newborns' care. On the contrary, many placed high trust in their healthcare providers. This could be due to many reasons. P~~The p~~arents may not feel ~~they were~~ sufficiently informed to make the -decision, hence leaving it up to the healthcare providers to decide what is best for the newborn. The~~ or they~~ parents reported that they trusted the healthcare providers were providing the best possible treatment. There is a need for ~~, or the local culture does not normally encourage such practice which makes parents unaware of the option to inquire and contribute to the discussion. F~~further research on the parentals~~'~~ involvement in decision making ~~should be conducte~~in the NCU and other healthcare settings~~d~~.

Formatted: Font: (Default) Times New Roman, Bold

Field Code Changed

Formatted: Font: (Default) Times New Roman

Field Code Changed

Formatted: Font: (Default) Times New Roman

Field Code Changed

Formatted: Font: (Default) Times New Roman

Having non-restrictive access to their newborns was one of the positive experiences for mothers. Such access, however, is not always allowed in many NCUs [23] and even other NCUs in Rwanda have restricted access [35]. Limited access to the NCUs ~~by~~ for fathers and other family members or caregivers was a source of concern for the parents in our study. Restricted NCU access is usually justified as necessary for infection control, ~~. H~~ however, studies ~~have present~~ ~~conflicting~~ controversial ~~results~~evidence. One study in the United Kingdom~~UK~~ found that restricted access was associated with a significant decline in respiratory infections [36], while another study in India showed that allowing parents in the NCU to directly participate in their newborn's care did not increase in hospital acquired infections [37]. NCU access restrictions have particularly been adopted during the COVID-19 pandemic where Neonatal Intensive Care Units that preserved the 24/7 parental presence decreased from 83 to 53% according to a global survey [38]. In all cases, education on hygiene practices are essential for all parents, visitors and providers. In the instances when the parents' full access to the NCU is scientifically justified as harmful to the newborn's health, an open and ongoing communication, emotional support, and the discussion on keeping the newborn connected to parents is needed [39].~~.~~

Little involvement of ~~The exclusion of fathers i~~fathers in the newborn care has~~n~~ particularly been ~~was a~~ source of ~~common~~ negative experience ~~shared by our study participants~~for mothers and fathers. ~~Such exclusion~~The lack of father involvement in the newborn care could reduce father's role to merely providing financial support. Studies have shown ~~this~~ that little or no involvement of fathers in NCU ~~could leave~~leave the fathers ~~feeling~~ scared of ~~their~~ their preterm newborns, affect early bonding, and father-child relationships [3,40]. Recognizing the importance of parental access to newborns, some healthcare providers break the

Formatted: Font: (Default) Times New Roman

Field Code Changed

Formatted: Font: (Default) Times New Roman

Field Code Changed

Field Code Changed

Formatted: Font: (Default) Times New Roman

Formatted: Font: (Default) Times New Roman

Field Code Changed

Commented [SB3]: 1. Darcy Mahoney A, White RD, Velasquez A, Barrett TS, Clark RH, Ahmad KA. Impact of restrictions on parental presence in neonatal intensive care units related to coronavirus disease 2019. *J Perinatol*. 2020;40(1):36-46. doi:10.1038/s41372-020-0753-7

Commented [SB4]: 1. Murray PD, Swanson JR. Visitation restrictions: is it right and how do we support families in the NICU during COVID-19? *J Perinatol*. 2020;40(10):1576-1581. doi:10.1038/s41372-020-00781-1

Field Code Changed

Formatted: Font: (Default) Times New Roman

rules and allowed occasional visitations – leading to some reports of “good nurses” and “bad nurses” [41]. Hospitals should reconsider the NCU visitation policy, and aim to deliver family-centered care to promote the best experience for the families and their newborns.

The quality of communication between caregivers and providers greatly impacted the experience of parents in the NCU. When NCU staff provided information about the newborn’s condition and showed caring attitude, parents felt more hopeful, understanding, confident and a will to trust healthcare providers. Similar to the literature, our study highlighted that poor communication and blame by healthcare providers are a source of stress for parents [42].

The parental interactions and peer support were a source of positive experience for parents and should be encouraged. A systematic review has shown that informal or formalized peer support could improve the experiences and well-being of parents [43]. Such findings further reinforced the importance of clear communication from healthcare providers. When healthcare providers gave parents proper and clear orientation and instructions, such messages will be passed along among other parents.

Almost all respondents in our study mentioned the cost was a great source of stress. In Rwanda, 78.5% of the population were enrolled in the Community Based Health Insurance (CBHI) in 2019 [44]. Basic drugs and medical services are covered by CBHI. However, patients or their families are often required to pay for more specialized services and diagnostics. And if medication stock out occurs, they have to purchase the medications for outside the hospitals. All these can contribute to extra financial burden. Additionally, indirect medical cost like transportation, food, and time from work are all imposing significant financial cost to the families. Similar to other studies, the cost of the NCU care, materials needed for the care, transportation, and food are serious challenges [45]. Studies to assess the cost related to NCU

Formatted: Font: (Default) Times New Roman

Field Code Changed

Formatted: Font: (Default) Times New Roman

Field Code Changed

Field Code Changed

Formatted: Font: (Default) Times New Roman

Formatted: Not Highlight

Formatted: Not Highlight

Formatted: Not Highlight

Field Code Changed

Formatted: Font: (Default) Times New Roman

admission, and potential catastrophic out of pocket expenditures, should be investigated in order to inform policy makers if advocacy for expanding insurance coverage is needed.

Limitations

Formatted: Font: 16 pt, Bold

Formatted: Indent: First line: 0"

This study has some limitations. Most respondents in our study were women, and insights from fathers were relatively limited. The current study was conducted in a district hospital that receives support from a few non-government organizations. Not all Rwandan hospitals receive such support, thus the findings may not be generalizable to other Rwandan health facilities.

Conclusion

Formatted: Font: (Default) Times New Roman, Bold

The admission of a newborn into the NCU is a source of stress for parents. Their stress is exacerbated by the environment including barriers to access by fathers and other family members, high-cost of neonatal care, and negative interactions with healthcare providers. However, the experience of care is improved by unrestricted access to the newborns by mothers as well as good communication with and trust of healthcare providers. Health facilities should also consider expanding the NCU access, encouraging peer support and actively involving parents in the care of their newborns. Further work is needed to understand the financial barriers for accessing NCU care.

Formatted: Font: Times New Roman, Font color: Auto

Acknowledgements

Formatted: Font: 18 pt

The authors are grateful to Ruli District hospital administration and Neonatal Care Unit staff, for their support to allow this study to be conducted. We thank the parents who participated for taking the time to share their story.

Formatted: Indent: First line: 0.5"

List of ~~abreviations~~abbreviations

Formatted: Font: Bold

- ABC: All Babies Count
- LMIC: Low- and Middle-Income Countries
- NCU: Neonatal Care Unit
- RNEC: Rwanda National Ethics Committee
- PTSD: Post-Traumatic Stress Disorder

Declarations

Formatted: Font: Bold

Ethics approval and consent to participate

Formatted: Font: 16 pt, Not Italic

Formatted: Font: 16 pt

The study was approved by the Rwanda National Ethics Committee (No. 107/RNEC/2019), University of Global Health Equity and Ruli District Hospital. Signed written consents were obtained from all participants.

Consent for publication

Formatted: Font: 16 pt, Not Italic

Formatted: Font: 16 pt

All co-authors read and approved the final manuscript.

Availability of data and materials

Formatted: Font: 16 pt, Not Italic

Formatted: Font: 16 pt

Not applicable

Competing interests

Formatted: Font: 16 pt, Not Italic

Formatted: Font: 16 pt

The authors declare that they have no competing interests.

Funding

Formatted: Font: 16 pt, Not Italic

Formatted: Font: 16 pt

The study was conducted with support from Grand Challenges Canada Saving Lives at Birth as part of the evaluation of the All Babies Count initiative. UGHE covered the cost of Dedoose software utilized in the analysis.

Authors' contributions

Formatted: Font: 16 pt, Not Italic

Formatted: Font: 16 pt

SB contributed to the study design, data collection, analysis, and writing the manuscript.

CK and RW contributed in the study design, data analysis and writing the manuscript.

JL contributed in writing the manuscript.

DK, SU, and JG contributed to the review of the manuscript.

~~Acknowledgements~~

Formatted: Font: 16 pt, Not Italic

Formatted: Font: 16 pt

~~The authors are grateful to Ruli District hospital administration and Neonatal Care Unit staff, for their support to allow this study to be conducted. We thank the parents who participated for taking the time to share their story.~~

References

1. WHO. Newborns: reducing mortality [Internet]. World Heal. Organ. 2018 [cited 2019 Dec 11]. p. 2017–20. Available from: https://www.who.int/news-room/fact-sheets/detail/newborns-reducing-mortality

2. Bhutta ZA, Das JK, Bahl R, Lawn JE, Salam RA, Paul VK, et al. Can available interventions end preventable deaths in mothers, newborn babies, and stillbirths, and at what cost? Lancet [Internet]. Elsevier; 2014;384:347–70. Available from: https://doi.org/10.1016/S0140-6736(14)60792-3

3. Adama EA, Sundin D, Bayes S. Ghanaian fathers' experiences of caring for preterm infants; a journey of exclusion. J Neonatal Nurs [Internet]. Elsevier Ltd; 2017;23:275–81. Available from: https://doi.org/10.1016/j.jnn.2017.05.003

4. EFCNI. Welcoming the family in the neonatal intensive care unit [Internet]. 2016. Available from: https://www.efcni.org/wp-content/uploads/2018/03/2016_05_02_Factsheet_A4_Welcoming_the_family_mE.pdf

5. Heidari H, Hasanpour M, Fooladi M. The experiences of parents with infants in Neonatal Intensive Care Unit. Iran J Nurs Midwifery Res [Internet]. Medknow Publications & Meida Pvt Ltd; 2013;18:208–13. Available from: https://www.ncbi.nlm.nih.gov/pubmed/23983756

6. Ncube RK, Barlow H, Mayers PM. A life uncertain – My baby's vulnerability: Mothers' lived experience of connection with their preterm infants in a Botswana neonatal intensive care unit. Curationis [Internet]. 2016 [cited 2019 Dec 6];39:9. Available from: http://www.curationis.org.za/index.php/curationis/article/view/1575

7. Obeidat HM, Bond EA, Callister LC. The Parental Experience of Having an Infant in the Newborn Intensive Care Unit. J Perinat Educ [Internet]. United States: Lamaze International;

Formatted: Font: 18 pt

2009;18:23–9. Available from:
http://www.ingentaconnect.com/content/lamaze/jpe/2009/00000018/00000003/art00006
8. Steyn E, Poggenpoel M, Myburgh C. Lived experiences of parents of premature babies in the intensive care unit in a private hospital in Johannesburg, South Africa. Curationis [Internet]. AOSIS Publishing; 2017 [cited 2019 Dec 6];40:1–8. Available from: http://www.curationis.org.za/index.php/curationis/article/view/1698
9. Dunn MS, Reilly MC, Johnston AM, Hoopes RD, Abraham MR. Development and Dissemination of Potentially Better Practices for the Provision of Family-Centered Care in Neonatology: The Family-Centered Care Map. Pediatrics [Internet]. 2006;118:S95 LP-S107. Available from: http://pediatrics.aappublications.org/content/118/Supplement_2/S95.abstract
10. American Academy of Pediatrics. Patient- and Family-Centered Care and the Pediatrician's Role. Pediatrics [Internet]. 2012;129:394 LP – 404. Available from: http://pediatrics.aappublications.org/content/129/2/394.abstract
11. Hagen IH, Iversen VC, Nesset E, Orner R, Svindseth MF. Parental satisfaction with neonatal intensive care units: a quantitative cross-sectional study. BMC Health Serv Res [Internet]. BioMed Central; 2019 [cited 2019 Dec 6];19:37. Available from: https://bmchealthservres.biomedcentral.com/articles/10.1186/s12913-018-3854-7
12. Bernard-Bonnin A-C. Maternal depression and child development. Paediatr Child Health [Internet]. Pulsus Group Inc; 2004 [cited 2018 Feb 24];9:575–83. Available from: https://academic.oup.com/pch/article/9/8/575/2648627
13. Guimarães H. The importance of parents in the neonatal intensive care units. J Pediatr Neonatal Individ Med [Internet]. 2015 [cited 2019 Dec 6];4:e040244. Available from: http://www.jpnim.com/index.php/jpnim/article/view/040244
14. Lefkowitz D, Baxt C, Evans J. Prevalence and Correlates of Posttraumatic Stress and

Postpartum Depression in Parents of Infants in the Neonatal Intensive Care Unit (NICU). J Clin Psychol Med Settings [Internet]. Boston: Springer US; 2010;17:230–7. Available from: https://www.ncbi.nlm.nih.gov/pubmed/20632076

15. Poehlmann J, Schwichtenberg AM, Bolt D, Dilworth-Bart J. Predictors of Depressive Symptom Trajectories in Mothers of Preterm or Low Birth Weight Infants. J Fam Psychol [Internet]. NIH Public Access; 2009 [cited 2018 Feb 24];23:690–704. Available from: http://www.ncbi.nlm.nih.gov/pubmed/19803605

16. Rogers CE, Kidokoro H, Wallendorf M, Inder TE. Identifying mothers of very preterm infants at-risk for postpartum depression and anxiety before discharge. J Perinatol [Internet]. 2013 [cited 2019 Dec 6];33:171–6. Available from: http://www.nature.com/articles/jp201275

17. Black MM, Walker SP, Fernald LCH, Andersen CT, Digirolamo AM, Lu C, et al. Advancing Early Childhood Development: from Science to Scale 1: Early childhood development coming of age: science through the life course [Internet]. Lancet (London, England). 2016. p. 77–90. Available from: https://www.openaire.eu/search/publication?articleId=od_______267::06e6dfa66cc543657f622320235886d7

18. World Bank. World Bank Country and Lending Groups – World Bank Data 2019-2020 [Internet]. World Bank. 2019 [cited 2019 Dec 6]. p. 1–8. Available from: https://datahelpdesk.worldbank.org/knowledgebase/articles/906519-world-bank-country-and-lending-groups

19. Rwanda 2014-15. Stud Fam Plann [Internet]. John Wiley & Sons, Ltd (10.1111); 2016 [cited 2019 Dec 6];47:395–404. Available from: http://doi.wiley.com/10.1111/sifp.12002

20. Hansen A, Magge H, Labrecque M, Munyaneza RBM, Nahimana E, Nyishime M, et al. The development and implementation of a newborn medicine program in a resource-limited setting.

Public Heal action [Internet]. 2015;5:2220-8372:17. Available from: http://www.ncbi.nlm.nih.gov/pubmed/26400597%5Cnhttp://www.pubmedcentral.nih.gov/articlerender.fcgi?artid=PMC4525367

21. Charpak N, Tessier R, Ruiz JG, Hernandez JT, Uriza F, Villegas J, et al. Twenty-year Follow-up of Kangaroo Mother Care Versus Traditional Care. Pediatrics [Internet]. 2017 [cited 2019 Dec 6];139:e20162063. Available from: http://www.ncbi.nlm.nih.gov/pubmed/27965377

22. Karlsson V, Heinemann A-B, Sjörs G, Nykvist KH, Ågren J. Early Skin-to-Skin Care in Extremely Preterm Infants: Thermal Balance and Care Environment. J Pediatr [Internet]. 2012 [cited 2019 Dec 6];161:422–6. Available from: http://www.ncbi.nlm.nih.gov/pubmed/22497906

23. Musabirema P, Brysiewicz P, Chipps J. Curationis. [Internet]. Curationis. DENOSA; 2015 [cited 2018 Feb 24]. Available from: https://curationis.org.za/index.php/curationis/article/view/1499/1874#2

24. Tunçalp Ö, WM W, MacLennan C, OT O, AM G, Bahl R, et al. Quality of care for pregnant women and newborns—the WHO vision. BJOG An Int J Obstet Gynaecol [Internet]. Wiley/Blackwell (10.1111); 2015;122:1045–9. Available from: https://doi.org/10.1111/1471-0528.13451

25. Hawell E, Graham C. Parents ' experiences of neonatal care: A report on the findings from a national survey. Pick Inst Eur. 2011;1–2.

26. O'Brien K, Robson K, Bracht M, Cruz M, Lui K, Alvaro R, et al. Effectiveness of Family Integrated Care in neonatal intensive care units on infant and parent outcomes: a multicentre, multinational, cluster-randomised controlled trial. Lancet Child Adolesc Heal. 2018;2:245–54.

27. Rwanda Ministry of Health. Rwanda health management information system. 2017.

28. Rwanda Ministry of Health. Neonatology: Clinical Treatment Guidelines [Internet]. Kigali; 2012. Available from: www.moh.gov.rw

29. MOH Rwanda. Health Service Packages for Public Health Facilities • Rwanda Healthcare System • [Internet]. 2017. Available from: www.moh.gov.rw

30. Nyishime M, Borg R, Ingabire W, Hedt-Gauthier B, Nahimana E, Gupta N, et al. A retrospective study of neonatal case management and outcomes in rural Rwanda post implementation of a national neonatal care package for sick and small infants. BMC Pediatr [Internet]. BioMed Central; 2018 [cited 2020 Oct 15];18:353. Available from: https://bmcpediatr.biomedcentral.com/articles/10.1186/s12887-018-1334-1

31. Ruzibiza Y. 'They are a shame to the community … ' stigma, school attendance, solitude and resilience among pregnant teenagers and teenage mothers in Mahama refugee camp, Rwanda. Glob Public Health [Internet]. Routledge; 2020 [cited 2021 Feb 16]; Available from: https://doi.org/10.1080/17441692.2020.1751230

32. Ottosson C, Lantz B. Parental participation in neonatal care. J Neonatal Nurs. 2017;23:112–8.

33. Tandberg BS, Sandtrø HP, Vårdal M, Rønnestad A. Parents of preterm evaluation of stress and nursing support. J Neonatal Nurs. 2013;19:317–26.

34. Østergaard LR. Trust matters: A narrative literature review of the role of trust in health care systems in sub-Saharan Africa. Glob Public Health [Internet]. Taylor & Francis; 2015;10:1046–59. Available from: https://doi.org/10.1080/17441692.2015.1019538

35. Agaba F, Kalimba E, Khadka N, Mwali K, Shelus V, Wisner L. Situation Analysis of Inpatient Care of Newborns and Young Infants Authors : Matern. Child Surviv. Progr. Washington (DC); 2019.

36. Lakshmanan A, Szatkowski L, Zinna S, Clarkson M, McClaughry R, Soo S, et al. G109 Restricting visitors to the nicu significantly reduces nosocomial viral respiratory tract infections in babies. Arch Dis Child [Internet]. 2015;100:A47 LP-A47. Available from:

http://adc.bmj.com/content/100/Suppl_3/A47.1.abstract
37. Verma A, Maria A, Pandey RM, Hans C, Verma A, Sherwani F. Family-centered care to complement care of sick newborns: A randomized controlled trial. Indian Pediatr [Internet]. 2017 [cited 2019 Dec 6];54:455–9. Available from: http://www.ncbi.nlm.nih.gov/pubmed/28667715
38. Darcy Mahoney A, White RD, Velasquez A, Barrett TS, Clark RH, Ahmad KA. Impact of restrictions on parental presence in neonatal intensive care units related to coronavirus disease 2019. J Perinatol [Internet]. Springer Nature; 2020 [cited 2021 Feb 19];40:36–46. Available from: https://www.nature.com/articles/s41372-020-0753-7
39. Murray PD, Swanson JR. Visitation restrictions: is it right and how do we support families in the NICU during COVID-19? J Perinatol [Internet]. Springer Nature; 2020 [cited 2021 Feb 19];40:1576–81. Available from: https://www.nature.com/articles/s41372-020-00781-1
40. Fisher D, Khashu M, Adama EA, Feeley N, Garfield CF, Ireland J, et al. Fathers in neonatal units: Improving infant health by supporting the baby-father bond and mother-father coparenting. J Neonatal Nurs [Internet]. Elsevier; 2018 [cited 2019 Dec 6];24:306–12. Available from: https://www.sciencedirect.com/science/article/abs/pii/S1355184118300930
41. Griffin T. A Family-Centered "Visitation" Policy in the Neonatal Intensive Care Unit That Welcomes Parents As Partners. J Perinat Neonatal Nurs [Internet]. 2013;27. Available from: https://journals.lww.com/jpnnjournal/Fulltext/2013/04000/A_Family_Centered__Visitation__Policy_in_the.12.aspx
42. Williams KG, Patel KT, Stausmire JM, Bridges C, Mathis MW, Barkin JL. The neonatal intensive care unit: Environmental stressors and supports. Int J Environ Res Public Health. 2018;15.
43. Altimier L. Newborn and Infant Nursing Reviews. Newborn Infant Nurs Rev [Internet]. W.B. Saunders; 2009 [cited 2019 Dec 6];9:129. Available from:

https://www.sciencedirect.com/science/article/pii/S1527336909000841

44. Rwanda Social Security Board. Community-Based Health Insurance Fiscal Year 2018/2019 Annual Report. 2019.

45. Martinez AM, Khu DTK, Boo NY, Neou L, Saysanasongkham B, Partridge JC. Barriers to neonatal care in developing countries: Parents' and providers' perceptions. J Paediatr Child Health. 2012;48:852–8.

Supporting information

S1 File. Semi-structured interview guide (English).

S2 File. Semi-structured interview guide (Kinyarwanda)

Formatted: Font: 12 pt

Formatted: Font: 12 pt

Formatted: Font: 12 pt
