# Supplementary material for: A qualitative study to explore the experience of parents of newborns admitted to neonatal care unit in rural Rwanda
Source: PLoS One. 2021 Aug 13;16(8):e0252776. doi: 10.1371/journal.pone.0252776 (PMC8362984; doi:10.1371/journal.pone.0252776)
Supplement: Attachment — Submitted filename: Response to Reviewers.docx [file pone.0252776.s004.docx]

Dear Editor,

We have addressed the comments from you and the reviewers as follows:

- Reviewer #2: Overall the paper has been much improved. It still requires further language editing. Trustworthiness/rigor is still not discussed. I could not find the COREQ list.The authors have still written from a quantitative perspective - e.g. they refer to generalisability which would not apply in a small qualitative study.
  - We added the rigor/trustworthiness statements in both the analysis and data collection sections.
  - We added the COREQ checklist in the supplementary files
  - We changed the generalizability to transferability of the findings
  - We did the language editing according to the recommendations given by the editor
- Additional changes
  - We removed the ethics statement from the declaration section.
  - To answer all relevant questions asked by the COREQ, we added the average duration of interviews under the results section,
  - We included a statement which explains why mothers reported tiredness looking after their babies day and night – according to policy at the study site on page 16
  - We did language editing on page 25 to clarify why patients still suffer the out of pocket burden while covered by the Community Based Health Insurance.
  - Reviewed the references to ensure they are all accessible
